# Vaccine adjuvants for infectious disease in the clinic

**DOI:** 10.1002/btm2.10663

**Published:** 2024-03-22

**Authors:** Morgan Goetz, Naaz Thotathil, Zongmin Zhao, Samir Mitragotri

**Affiliations:** ^1^ John A Paulson School of Engineering & Applied Sciences Harvard University Allston Massachusetts USA; ^2^ Wyss Institute of Biologically Inspired Engineering Boston Massachusetts USA; ^3^ University of Massachusetts Amherst Amherst Massachusetts USA; ^4^ Department of Pharmaceutical Sciences College of Pharmacy, University of Illinois Chicago Chicago Illinois USA

**Keywords:** Adjuvants, clinic, clinical trials, FDA, immune response, infectious diseases, vaccines

## Abstract

Adjuvants, materials added to vaccines to enhance the resulting immune response, are important components of vaccination that are many times overlooked. While vaccines always include an antigen to tell the body what to vaccinate to, of equal importance the adjuvant provides the how, a significant factor in producing a complete response. The adjuvant space has been slow to develop with the first use of an adjuvant in a licensed vaccine occurring in the 1930s, and remaining the only adjuvant in licensed vaccines for the next 80 years. However, with vaccination at the forefront of protection against new and complex pathogens, it is important to consider all components when designing an effective vaccine. Here we summarize the adjuvant space in licensed vaccines as well as the novel adjuvant space in clinical trials with a specific focus on the materials utilized and their resulting impact on the immune response. We discuss five major categories of adjuvant materials: aluminum salts, nanoparticles, viral vectors, TLR agonists, and emulsions. For each category, we delve into the current clinical trials space, the impact of these materials on vaccination, as well as some of the ways in which they could be improved. Adjuvants present an exciting opportunity to improve vaccine responses and stability, this review will help inform about the current progress of this space.

**Translational impact statement:**

In the aftermath of the COVID‐19 pandemic, vaccines for infectious diseases have come into the spotlight. While antigens have always been an important focus of vaccine design, the adjuvant is a significant tool for enhancing the immune response to the vaccine that has been largely underdeveloped. This article provides a broad review of the history of adjuvants and, the current vaccine adjuvant space, and the progress seen in adjuvants in clinical trials. There is specific emphasis on the material landscape for adjuvants and their resulting mechanism of action. Looking ahead, while the novel vaccine adjuvant space features exciting new technologies and materials, there is still a need for more to meet the protective needs of new and complex pathogens.

## 1 INTRODUCTION

Vaccination is an important public health strategy against communicable diseases. Vaccines train the immune system using an antigen, thereby allowing the body to generate an immune response in the form of antigen‐specific antibodies and/or antigen‐specific T‐cells. Antigen and adjuvant are two essential components of a vaccine. Antigens convey the immune system ‘what’ to vaccinate against, while adjuvants, derived from the Latin word adjuvare meaning “to aid”, boost the magnitude of the immune response. Adjuvants can also render additional benefits including reduction of the required antigen dose, which provide cost and compliance benefits.

Adjuvants have been prevalent in vaccines since the discovery of aluminum salt (alum) as an adjuvant in 1926 by Alexander T. Glenny.^1^ It was utilized in the diphtheria and tetanus vaccines licensed by the U.S. Food and Drug Administration (FDA) in the 1930s. Since then, alum has been added to numerous vaccines to enhance the immune response. However, despite sparse research, no additional adjuvants beyond alum were utilized in licensed vaccines until 2009.^2^ With this paucity of adjuvant materials, many vaccines were designed utilizing live‐attenuated viruses (e.g., MMR and Chickenpox vaccines) to take advantage of their innate adjuvanticity; however, they carry a risk for immunocompromised individuals.^3^ Additionally, many vaccines employed an inactivated (killed) pathogen without adjuvants (e.g., seasonal flu and polio vaccines) which can struggle to achieve efficacy alone.^4^ Due to these difficulties, as well as the advent of recombinant protein antigen in the 1980s, which are less immunogenic than both live‐attenuated and whole killed antigens, the importance of adjuvant innovation became more apparent.^5^ Since the 2010s, adjuvant innovation has been on the rise with several materials added to FDA licensed vaccines and many others reaching clinical studies, especially in the wake of several pandemics such as Ebola, Zika, and COVID‐19. Here, we provide an overview of the current clinical landscape of adjuvants for vaccination against communicable diseases. We highlight 12 adjuvants in FDA‐licensed vaccines and discuss >300 active clinical trials of vaccine adjuvants. Adjuvants in this review is broadly defined as the ingredient separate from the antigen that produces an immune response, which allows antigen carriers such as lipid nanoparticles and viral vectors to be evaluated although not acknowledged as an adjuvant by the CDC. The primary focus of this review is to provide the readers with the current clinical landscape of vaccine adjuvants, which may guide future efforts in developing safer and more effective adjuvants.

## 2 ADJUVANTS IN LICENSED VACCINES

Adjuvants employed in licensed vaccines have rapidly evolved since 2010s, before which aluminum salts was the only adjuvant in licensed vaccines. As our understanding of immunology and vaccinology alike has evolved, more materials with different mechanism of actions (MOA) have been developed. So far, 12 adjuvants have been utilized in vaccines licensed by the FDA (Table 1). Here, we review these products and highlight their indications.

**TABLE 1:** Adjuvants in FDA licensed vaccines.

Adjuvant	Licensure year	Composition	Vaccines
Aluminum	1930s	One or more of the following: aluminum hydroxide, aluminum phosphate, aluminum potassium sulfate (alum), amorphous aluminum hydroxyphosphate sulfate (AAHS)	Anthrax, DT, DTaP (Daptacel, Infanrix), DTaP‐HepB‐IPV (Pediarix), DTaP‐IPV (Kinrix, Quadracel), DTaP –IPV/Hib (Pentacel), DTaP‐IPV‐Hib‐HepB (VAXELIS), HepA (Havrix, Vaqta), HepB (Engerix‐B, PREHEVBRIO, Recombivax), HepA/HepB (Twinrix), HIB (PedvaxHIB), HPV (Gardasil 9), Japanese encephalitis (Ixiaro), MenB (Bexsero, Trumenba), Pneumococcal (Prevnar 13, Prevnar 20, VAXNEUVANCE), Td (Tenivac, Mass Biologics), Tdap (Adacel, Boostrix), Tick‐Borne Encephalitis (TICOVAC)
AS04	2009	Monophosphoryl lipid A (MPL) absorbed on aluminum hydroxide	HPV Vaccine (Cervarix)
AS03	2013	Oil in water emulsion of (d,l)‐α‐tocopherol, squalene, and tween 80	H1N1 Vaccine—*Currently in US Stockpile*
MF59	2015	Oil in water emulsion of squalene, span 85, and tween 80	Seasonal influenza vaccine (Fluad and Fluad Quadrivalent)
Outer Membrane Vesicle (OMV)	2015	Outer membrane vesicle derived from N. Meningitidis	Group B meningococcus (Bexsero)
AS01_B_	2017	Liposomal formulation of QS‐21 and MPL	Zoster vaccine (Shingrix)
CpG 1018	2017	22‐mer oligonucleotide sequence containing CpG motifs	HepB (Heplisav‐B)
rVSV	2019	Live attenuated recombinant vesicular stomatitis virus (rVSV) vector	Ebola Vaccine (Ervebo)
BioNTech Lipid Nanoparticle	2021	4‐hydroxybutyl)azanediyl)bis(hexane‐6,1‐diyl)bis(2‐hexyldecanoate), 2‐[(polyethylene glycol)‐2000]‐*N*,*N*‐ditetradecylacetamide, 1,2‐distearoyl‐*sn*‐glycero‐3‐phosphocholine [DSPC], and cholesterol	COVID‐19 Vaccine (Comirnaty)
Moderna Lipid Nanoparticle	2022	SM‐102, polyethylene glycol [PEG], 2000 dimyristoyl glycerol [DMG], 1,2‐distearoyl‐sn‐glycero‐3‐phosphocholine [DSPC], and cholesterol	COVID‐19 Vaccine (Spikevax)
MatrixM (EUA)	2022	Nanoparticle formulation of saponin fractions resulting in Matrix‐A and Matrix C delivered together as Matrix‐M	COVID‐19 vaccine (Novavax)
CpG 7909	July 2023	24‐mer oligonucleotide sequence containing CpG motifs	Anthrax Vaccine (Cyfendus)

### 2.1 Aluminum salts

**FIGURE 1:**
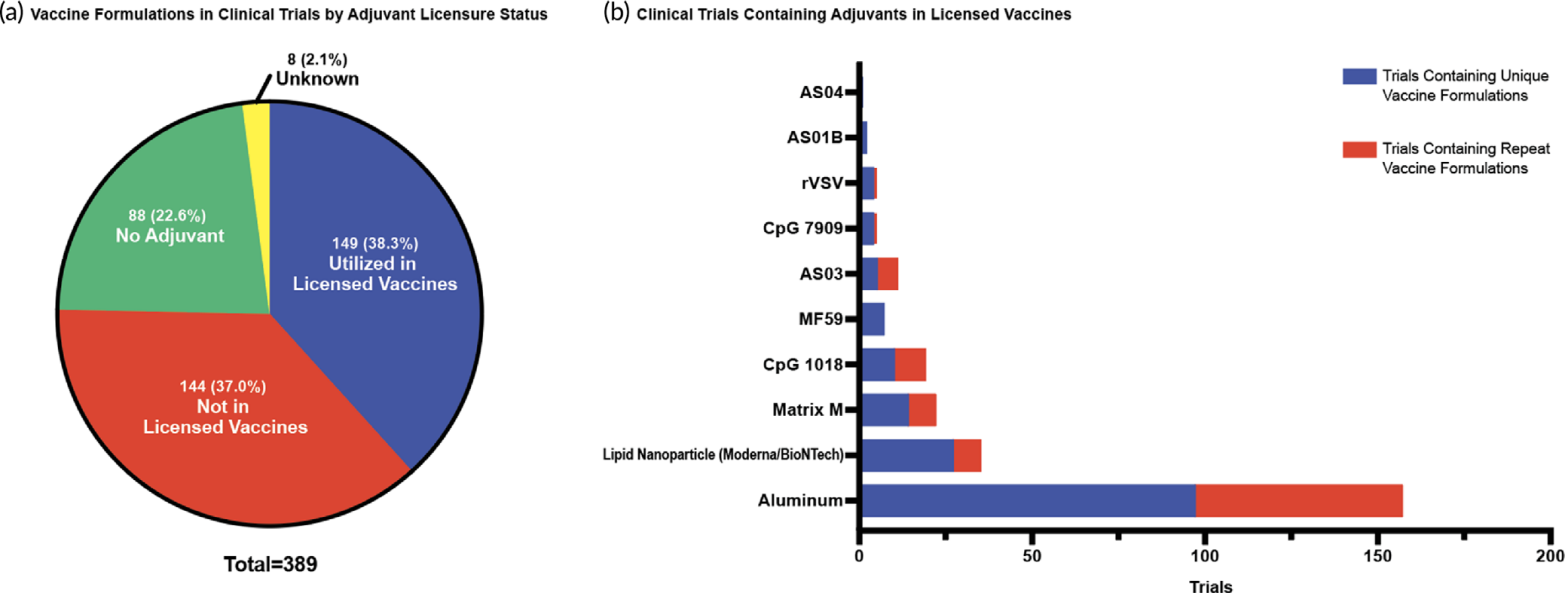
Adjuvants in licensed vaccines in the current clinical trial space. (a) The classification of unique vaccine formulations evaluated in clinical trials by adjuvant licensure status in which a unique vaccine formulation is defined by a distinct combination of adjuvant and antigen among the trial space. Each section displays the unique vaccine formulation count and that percentage of the whole. (b) The number of clinical trials that contain an adjuvant in a licensed vaccine displayed as the number of trials in which this adjuvant is utilized in a unique vaccine formulation and the number of trials in which the adjuvant is utilized in a repeat vaccine formulation.

Aluminum salts in its various forms have dominated the adjuvant space since the 1930s. It was originally discovered by accident when Glenny purified diphtheria toxoids with aluminum potassium sulfate and found that vaccines with aluminum salt precipitates led to a stronger antibody response.^6^ Currently there are four types of aluminum salts utilized in vaccines—aluminum hydroxide (AH), aluminum phosphate (AP), aluminum potassium sulfate (alum), and amorphous aluminum hydroxyphosphate sulfate (AAHS)—with AH and AP being the most used in licensed vaccines. AH is prepared by mixing an aluminum solution with sodium hydroxide, resulting in crystalline aluminum oxyhydroxide.^7^ The degree of crystallinity impacts its absorption capacity, and AH composed of small crystals possess a larger surface area and stronger absorptive ability. AP is chemically amorphous aluminum hydroxyphosphate which is formulated with a solution of aluminum salt and a solution of trisodium phosphate or through mixing of aluminum salt and a phosphate solution and precipitating with sodium hydroxide.^8^, ^9^ Unlike AH, AP is noncrystalline resulting in a generally high absorption capacity dictated by buffer conditions and composition of starting materials. In addition to crystallinity, there also exists variations in the particle size and shape. AH present in the form of elongated particles with an average calculated dimension of 4.5 × 2.2 × 10 nm while AP are plate‐like particles of approximately 50 nm.^10^, ^11^ Despite their distinct physiochemical properties, because of their clinically validated efficacy and safety profiles, aluminum‐containing adjuvants have been used in many vaccines to specifically enhance the humoral response and continue to be utilized in active clinical trials (Table 1 and Figure 1b).

### 2.2 AS04

AS04 received approval from the FDA in 2009 and was developed by GlaxoSmithKline (GSK) for use in the HPV vaccine, Cervarix. This adjuvant still utilizes aluminum hydroxide as one of the key components, but it also adds in a toll‐like receptor (TLR) 4 agonist, monophosphoryl lipid A (MPL) to be absorbed onto the aluminum particulate. MPL is a purified and detoxified derivative of lipopolysaccharides (LPS). LPS is produced by many Gram‐negative bacteria and is present on their outer membrane, allowing them to be recognized by the immune system. Lipid A is the primary virulence factor of LPS, making it ideal to manipulate into an agonist.^12^ Specifically for AS04, the LPS that MPL is derived from is the R595 strain of *Salmonella minnesota*. In preclinical development, a 200 nm particle size was found to be ideal with 50 μg of MPL and 500 μg of aluminum hydroxide in each dose.^13^ The addition of MPL was shown to create a stronger and longer‐lasting immune response (cellular immune response in particular) compared to the vaccine formulated with just aluminum alone. This is largely due to the early immune response that MPL was able to induce through activation of antigen presenting cells (APCs) and an increase in inflammatory cytokines.^14^

### 2.3 AS03

AS03 was also developed by GSK for use in the H1N1 vaccine and was licensed for use in 2013; however, this vaccine is currently in the US Stockpile and AS03 is not in any vaccine received currently. This is the first adjuvant of its kind utilized in a licensed vaccine, which doesn't include aluminum in the formulation and belongs to a new category of adjuvants, oil‐in‐water emulsion. AS03 contains squalene (21.38 mg/ml), Tween 80 (9.72 mg/ml), and (d,l)‐α‐tocopherol (23.72 mg/ml) formulated in phosphate buffered saline (PBS). The resulting particle size for AS03 is around 150 nm, which is thought to aid in APC uptake.^15^ AS03 induces stronger antigen‐specific antibody and T‐cell responses when compared to aluminum salt, as well as higher levels of cytokines and immune cell recruitment to the draining lymph nodes.^16^ In regards to the formulation, (d,l)‐α‐tocopherol enhances the magnitude of the immune response induced by AS03, which will be discussed later in this review.

### 2.4 MF59

MF59 was utilized in the seasonal flu vaccine (Fluad and Fluad Quadrivalent) which was licensed by the FDA in 2015 after acquiring European licensure in 1997.^17^ Currently the MF59‐adjuvanted seasonal flu vaccine is only given to people of 65 years and older to compensate for their attenuated immune responses.^18^ This adjuvant was developed by Novartis and is also an oil‐in‐water emulsion similar to AS03. MF59 is composed of squalene (4.3%) and two surfactants, Tween 80 (0.5%) and Span 85 (0.5%), in citrate buffer with a resulting particle size of around 160 nm. MF59 can enhance both cellular and humoral immune responses by producing an immunocompetent environment at the injection site and is reliant on all three components of the formulation working together to produce the most robust immune response.^19^, ^20^

### 2.5 Outer membrane vesicle

OMVs are not classically defined as an adjuvant due to its carrier abilities; however, their inherent adjuvanticity is important in their addition to the formulation. An OMV is derived from the outer membrane of Gram‐negative bacteria, resulting in spherical vesicles around 25–250 nm that display many bacterial antigens and proteins that can function as TLR agonists.^21^ This technology is only licensed for use in a Meningococcal Group B vaccine, Bexsero, developed by Novartis and then acquired by GSK before approval in the US in 2015. Various Gram‐negative bacteria produce OMVs naturally in response to stress but can also be derived with a detergent extraction, which is utilized for Bexsero. In the case of Bexsero, the OMV is delivered with recombinant protein antigens and provides inherent antigen components itself, the major one being PorA since it is derived from the *N. meningitidis* bacteria. One major advantage of the OMVs is the presence of bacterial antigens simultaneously expressed with potent TLRs naturally present on them, which makes OMVs strong drivers of innate immune responses.^22^ Bexsero is also formulated with aluminum hydroxide for additional adjuvanticity.

### 2.6 AS01_B_

AS01_B_ is another combination adjuvant comprised of a liposomal formulation of the saponin QS‐21 and the TLR4 agonist MPL approximately 100 nm in size.^23^ This adjuvant is utilized in the zoster vaccine (Shringrix) licensed by the FDA in 2017, as well as in the WHO prequalified RTS,S Malaria Vaccine. While MPL is used previously in the AS04 adjuvant system, this is the first use of QS‐21—a fraction 21 saponin molecule extracted from the bark of the South American tree *Quillaja saponaria* Molina—in a licensed vaccine. QS‐21 has specifically been found to modulate the innate immune system^24^ leading to an enhancement of both antigen‐specific antibody responses and CD8 T cells.^25^ QS‐21 in liposomal formulation is still utilized to deliver numerous TLR agonists and will be discussed further in the TLR agonist section of this review. When formulated together, MPL and QS‐21 work synergistically to enhance immune responses to the antigen.^26^

### 2.7 CpG motifs (1018 and 7909)

Unmethylated CpG motifs, CpG 1018 and CpG 7909, are two TLR9 agonists utilized in previously licensed vaccines. CpG 1018 was first utilized in a licensed vaccine in 2017 for the Hep‐B vaccine, Heplisav‐B; CpG 7909 was then utilized after this in July 2023 in the licensed anthrax vaccine, Cyfendus. CpG motifs, comprised of a central unmethylated CG dinucleotide plus flanking regions, are derived from synthetic oligodeoxynucleotides (ODN) which mimic bacterial DNA. Bacterial DNA represents a potent pathogen‐associated molecular pattern (PAMP), which activates the innate immune system. During infection, the unmethylated CpG motifs present at high rate in bacterial DNA interact with TLR9 on immune cells and trigger a protective immune response.^27^, ^28^ Through synthetic replication of bacterial DNA and isolation of synthetic unmethylated CpG motifs that specifically trigger specific immune responses, CpG 1018 and 7909 are adjuvants with specific TLR9 activation abilities leading to a protective response that is specifically skewed towards the cellular mediated, or Th1, response.^29^ CpG motifs are often used in combination with aluminum adjuvants (e.g., in FDA licensed anthrax vaccine containing CpG 7909), as the combination of these two adjuvants results in potent cellular and humoral immunity.

### 2.8 rVSV

The Ebola Virus epidemic in West Africa (2013–2016) led to the first licensed viral vector vaccine. Vesicular Stomatitis Virus (VSV) is a viral vector‐based adjuvant utilized in in Merck's Ebola vaccine called ERVEBO (also known as V920, rVSVΔG‐ZEBOV‐GP or rVSV‐ZEBOV) which was licensed by the FDA in 2015. ERVEBO is a replication‐competent, live attenuated recombinant vesicular stomatitis virus (rVSV) vector with a glycoprotein gene of ZEBOV in replacement of the VSV glycoprotein G.^30^ VSV glycoprotein G allows for the attachment and entry into host cells, which is mimicked by the ZEBOV glycoprotein for a ZEBOV antigen‐specific immune response. rVSV is unique compared to other licensed adjuvant systems in that it encodes the antigen for expression on the surface of the vector as opposed to being admixed or bound. However, the resulting immune response is still dictated by the rVSV vector, independent of the antigen, indicating this technology is a potent immunomodulator alone. Specifically, the vaccine elicits an infection similar to VSV, leading to specific cellular and antibody responses against the viral protein, therefore protecting the person from future exposure.^31^ The vaccine resulted in 100% vaccine efficacy for a single dose, protecting over 5000 vaccinated individuals, as well as their unvaccinated contacts.^32^

### 2.9 Lipid nanoparticle

Two lipid nanoparticle‐based vaccines have been licensed by the FDA from Pfizer‐BioNtech and Moderna. The lipid nanoparticles in the Pfizer‐BioNtech COVID‐19 vaccine, now known as Comirnaty (BNT162b2), was the first COVID‐19 vaccine in the US, licensed by the FDA in August 2021. BNT162b2 was shown to have 95% efficacy in preventing COVID‐19 among 43,548 participants.^33^ The antigen of the vaccine, nucleoside‐modified RNA (modRNA) encoding the spike protein of the SARS‐CoV‐19 virus, is protected by the lipid nanoparticle formulated with four lipids: an ionizable lipid (((4‐hydroxybutyl)azanediyl)bis(hexane‐6,1‐diyl)bis(2‐hexyldecanoate)), a PEGylated lipid (2‐[(polyethylene glycol)‐2000]‐*N*,*N*‐ditetradecylacetamide), and two structural lipids (1,2‐distearoyl‐*sn*‐glycero‐3‐phosphocholine (DSPC) and cholesterol).^34^ The vaccine has demonstrated strong, antigen‐specific antibody responses, CD8+ and CD4+ T‐cell responses, as well as IFNγ production by immune cells upon vaccination.^35^ The other lipid nanoparticle is utilized in Moderna's SPIKEVAX COVID‐19 vaccine, also known as mRNA‐1273, which was authorized for emergency use by the FDA in December 2020 and then later licensed in January 2022. Similar to Pfizer‐BioNtech's vaccine, Moderna's SPIKEVAX showed 94.1% efficacy among 30,420 participants. Although it had similar local and systemic reactions such as headaches, fever, and fatigue, there are no safety concerns.^36^ The vaccine contains a messenger ribonucleic acid (mRNA) antigen encapsulated by a lipid nanoparticle comprised of four lipids: SM‐102, polyethylene glycol (PEG) 2000 dimyristoyl glycerol (DMG), cholesterol, and 1,2‐distearoyl‐sn‐glycero‐3‐phosphocholine (DSPC). Similar to BNT162b2, mRNA‐1273 induces CD4 T follicular helper, CD8+ and Th1 responses.^37^

### 2.10 Matrix‐M

The COVID‐19 vaccine NVX‐CoV2373 produced by Novavax, contains the nanoparticle adjuvant Matrix‐M which was granted emergency approval by the FDA in October 2022. It was since paused and reauthorized for emergency use with an updated vaccine that includes the spike protein from the SARS‐CoV‐2 Omicron variant. Currently, it has be reauthorized for emergency use in October 2023 by the FDA. The Matrix‐M adjuvant is a 40 nm saponin‐based nanoparticle adjuvant that is a mixture of two purified fractions from the bark of the Quillaja saponaria tree, in combination with cholesterol and phospholipids.^38^ The vaccine is formulated with SARS‐CoV2 spike protein trimer antigens formulated into 27.2 nm nanoparticles and combined with the Matrix‐M adjuvant to make the NVX‐CoV2372 vaccine.^39^, ^40^ In combination the vaccine has demonstrated to induce a robust Th1 type and T follicular helper cell (Tfh) response promoting neutralizing antibodies, as well as a CD4+ T cell and CD8+ T cell response. The vaccine has shown to be safe with mild to moderate side effects such as injection site pain, headaches and faitgue.^41^

## 3 CURRENT CLINICAL TRIALS

In this section, we provide a snapshot of the landscape of current clinical trials of new investigational vaccine adjuvants as of June 2023. We searched on the clinicaltrials.gov database using key words “vaccine” and “communicable diseases” and status of “recruiting, not yet recruiting, active, not recruiting, or enrolling by invitation”. We also specified the study type to be “Interventional Studies”. The returned entries (641 in total) were then manually screened to exclude vaccines that have already been licensed by the FDA or prequalified by the WHO and were seeking an extension of use to different populations/dosing schedules. After exclusion, a total of 523 trials were identified. From this set, the distinct vaccine formulations were analyzed to identify the adjuvant component. When a vaccine formulation was consistent across multiple trials, meaning the same antigen and adjuvant combination was used, these trials were grouped together as one. Conversely, when a trial was evaluating multiple vaccine formulations by utilizing multiple adjuvants and/or antigens, that trial was counted multiple times dependent on the number of vaccine formulations. This resulted in 389 unique vaccine formulations being evaluated.

A range of adjuvants are used in the identified active vaccine clinical trials (Figure 1). However, 23% of the identified trials didn't contain an adjuvant in the vaccine formulation (Figure 1a). About 50% of these formulations without adjuvants use a live‐attenuated virus as the antigen which has inherently high immunogenicity. However, these types of vaccines may have increased safety concerns especially in those who are immunocompromised. Of the other 50% vaccine trials without an adjuvant, most utilize an inactivated pathogen as the antigen. The use of an inactivated pathogen can improve vaccine safety but may reduce immunogenicity as the pathogen cannot replicate or enter immune cells to promote a robust immune response. Furthermore, without an adjuvant, these vaccine formulations completely rely on the body identifying the inactivated antigen as dangerous and producing an immune response. The annual seasonal flu vaccine utilizes inactivated influenza virus as the antigen without an adjuvant. These vaccines usually have a limited efficacy. For example, in the 2007–2008 flu season, the protection rate of the flu vaccine, Fluzone, was reported to be only 68%, emphasizing the need of adding an adjuvant to increase vaccine immunogenicity.^4^

**FIGURE 2:**
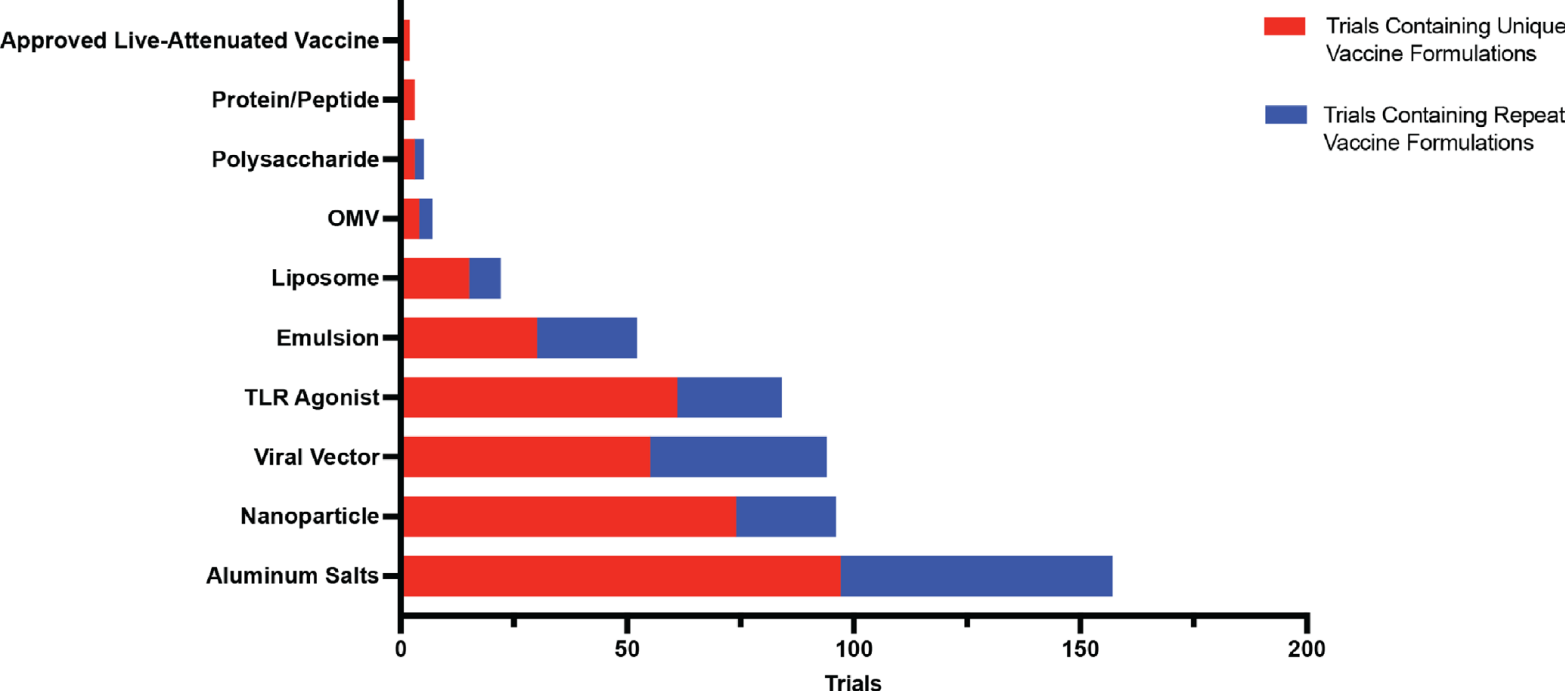
Number of clinical trials by adjuvant category. This is represented as both trials containing a unique vaccine formulation and those containing repeat formulations.

Some of the identified vaccine trials didn't specify the used adjuvant, and we classified these trials as “Unknown” in Figure 1a. In addition, for the vaccine formulations that did include adjuvants—previously utilized in licensed vaccines or novel—we further classified them based on different adjuvant categories (Figure 2). In the following discussions, we will step through these adjuvant categories, discuss their respective mechanism of actions, and overview the clinical trial landscape of each category.

### 3.1 Aluminum salts

Aluminum salts continue to dominate in the identified clinical trials representing the most pervasive adjuvant used in vaccine formulations currently being evaluated (Figure 2). Many of these formulations, ~88%, utilized aluminum salt as the only adjuvant in the formulation. However, ~10% of the formulations include a TLR‐agonist as a combination. Similar to what is seen in AS04, adding a TLR agonist can enhance the immune response, particularly cellular immune which aluminum‐based adjuvants struggle to achieve alone. TLR agonist focused trials will be discussed more in depth later in this review. In the current clinical trial landscape, vaccines formulated with aluminum adjuvants are being utilized for protection against numerous infectious diseases including COVID‐19, HIV, cholera, pertussis, among others.

Despite their long history in the use as an adjuvant, aluminum salts' mechanism of action in promoting immune responses have not been completely elucidated. After its discovery, Glenny et al. proposed the depot effect as a mechanism of action for the adjuvant, suggesting that the creation of a depot of antigen could result in continued and slow antigen release.^42^ This mechanism was largely accepted by the field for many years, but recently this idea has been challenged by the observation that removing the alum depot from the injection site as early as 2 h after injection did not influence the antigen‐specific immune response.^43^ This directly contrasts an early study done that shows removal of the depot within 4 days after injection impacted the immune response, but not when removed 7 days after injection.^44^ Overall, it remains unclear how the depot formation and duration of the depot impact the overall adjuvanticity. In addition to the depot effect, other immunomodulatory properties of alum have been explored to paint a more complete mechanistic picture. Specifically, aluminum adjuvants have been shown to induce a considerable amount of necrosis at the injection site which leads to the release of DAMPs such as DNA and uric acid.^45^, ^46^ These DAMPs promote the infiltration and activation of immune cells which have ultimately been connected to downstream immune responses. Of the immune cells that infiltrate into the site, dendritic cells have been identified as crucial cells to alum's mechanism. Dendritic cells not only enhance antigen transport to the draining lymph node from the site of injection,^47^ but in response to alum they are activated and the abundance of the population is increased through monocyte differentiation into dendritic cells.^48^ While alum struggles to initiate a cellular immune response, it is a potent humoral immune response modulator by activating the complement cascade.^49^

Even without a complete mechanistic understanding, aluminum adjuvants are continually utilized extensively in licensed vaccines or those in active clinical trials (Figure 2). With the longest track record of safety among all adjuvants in licensed vaccines, aluminum adjuvants have been trusted additives for the last 80 years. While new materials are needed to expand the adjuvant space, aluminum adjuvants continue to dominate in the clinical studies.

### 3.2 Nanoparticles

**FIGURE 3:**
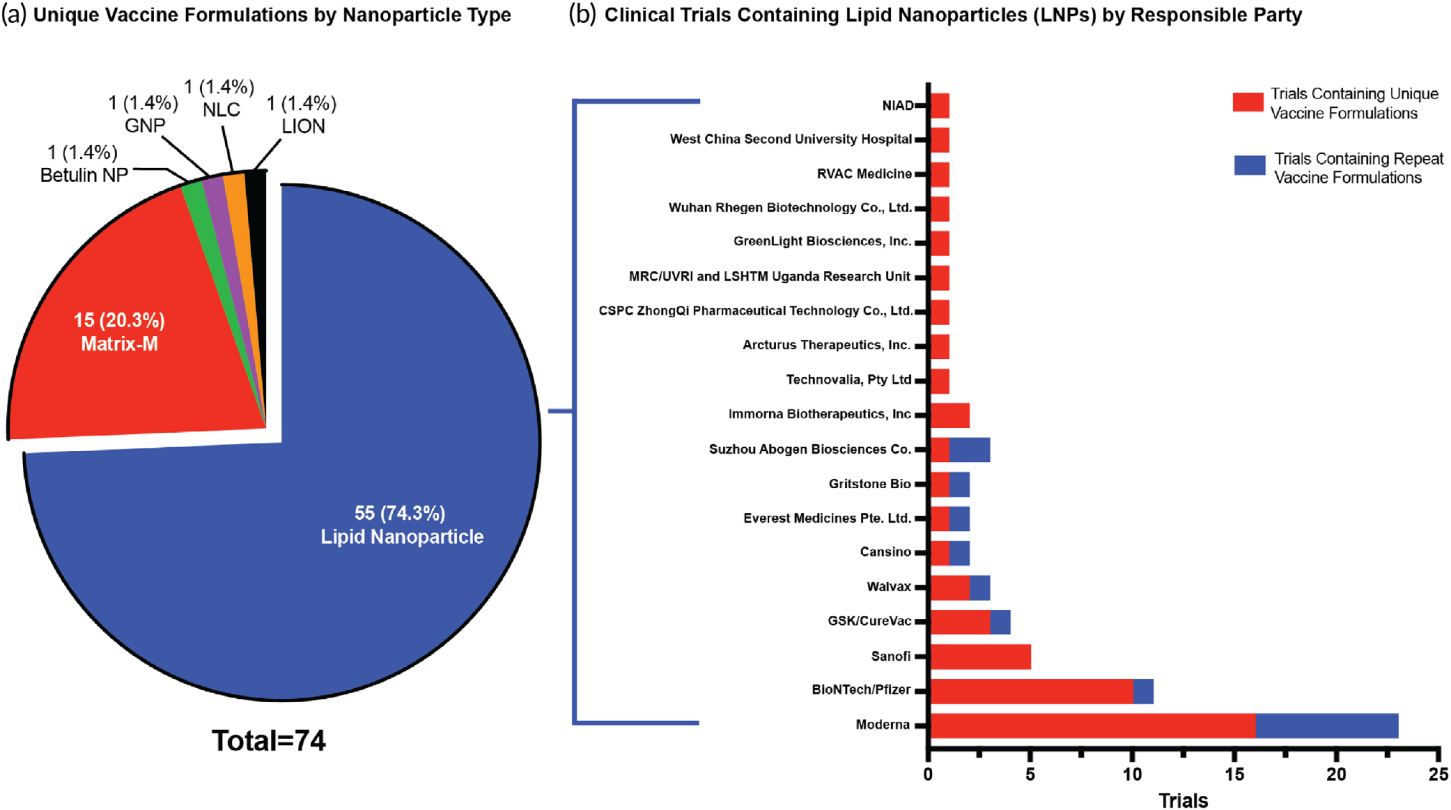
Nanoparticle adjuvants in the clinic. (a) Make‐up of unique vaccine formulations containing nanoparticle adjuvants in the clinical trial space separated by nanoparticle type: lipid nanoparticles, Matrix‐M, betulin nanoparticle (NP), gold nanoparticle (GNP), nanostructured lipid carriers (NLC), and lipid inorganic nanoparticles (LION). The number of unique vaccine formulations as well as the resulting percent of the whole space is provided. (b) The number of clinical trials that contain a lipid nanoparticle by responsible party of the clinical trial. This is defined by both number of trials with unique vaccine formulations and number of trials with repeat formulations.

Nanoparticles are another widely used vaccine adjuvant in the identified trials (Figure 2). Nanoparticles can be formulated with a range of materials and their physical and chemical properties such as size, shape, surface structure, physiochemistry, solubility, and hydrophobicity/hydrophilicity can be feasibly tuned. This has stimulated the use of nanoparticle adjuvants for vaccine development, especially since the COVID‐19 pandemic. While many are thought as just the antigen carrier, nanoparticles can function as adjuvants to enhance the immune response depending on their material and physicochemical properties. Nanoparticles are attractive candidates for use in vaccines due to their tunability. The antigen utilized can range from nucleic acids encapsulated inside the particle, to peptides and proteins formulated on the particle surface.^50^ The adjuvant component can also arise from the materials used to construct the particle, as in the case with Matrix M, or from ligands such as TLR agonists bound to the surface, including a potentially multi‐faceted immune response to the antigen.^51^ Some nanoparticles also allow for timed release, by utilizing gold material,^52^ which can enhance the immune response through frequent exposure. In the current clinical trial landscape, there are 75 unique vaccine formulations using nanoparticle‐based adjuvants with a total of 98 trials consisting of Matrix‐M, gold nanoparticles, lipid nanoparticles, and other variations of lipid carriers (Figure 3a). Among these, three nanoparticles have been previously utilized in licensed vaccines including the lipid nanoparticles designed by Pfizer‐BioNtech and Moderna and Matrix‐M designed by the University of Oxford. Nanoparticles have made a lot of progress among clinical vaccines since the COVID‐19 pandemic and continue to do so as the second largest adjuvant group in the identified active clinical trials, making up 18.2% of unique vaccine formulations and 16.4% of total trials (Figure 2).

#### 3.2.1 Lipid nanoparticles

Lipid nanoparticles (LNPs) are spherical vesicles made of varying ratios of ionizable lipids, helper lipid, cholesterol, and PEGylated lipid. By utilizing various materials in these roles and altering the ratios of them, many companies have produced their own LNPs for use in vaccination (Figure 3b). The composition of the four lipids encapsulates nucleic acids and proteins as antigen for the targeted disease. The ionizable lipid has a neutral charge at a physiological pH, reducing toxicity and facilitating the uptake of mRNA. When interacting with the cytoplasm, the induction of positive charge of the ionizable lipid induces the release of the mRNA into the host cell.^53^ LNPs make up the largest group of nanoparticle‐based adjuvants in the identified active clinical trials (Figure 3a), with 55 unique vaccine formulations spanning across 67 trials for vaccination against diseases such as COVID‐19, HIV, Shingles, Nipah Virus, and many more (Figure 3b). As mentioned earlier, there have been two licensed lipid nanoparticle vaccines: Moderna's SPIKEVAX COVID‐19 vaccine and Pfizer‐BioNTech's Comirnaty COVID‐19 vaccine. In a recent study, it was found that LNPs can induce strong humoral responses such as T‐Follicular helper (Tfh) cells, memory B‐cells, and plasma cells.^54^

The Pfizer‐BioNTech and Moderna vaccines have both demonstrated a similar mechanism of action. The vaccines contain nucleoside‐modified mRNA that encodes the viral spike glycoprotein of SARS‐CoV‐2‐S once entering the host cytoplasm, placed in a LNP for stability and protection.^55^ However, the LNP does not solely serve as an antigen carrier system, and instead has its own adjuvant abilities. Both BNT162b2 (NCT05541861) and mRNA‐1273 (NCT05383560) LNPs as well as the other LNPs utilized in the clinical trial space, contain the similar four key components—ionizable lipid, cholesterol, PEGylated lipid, and 1,2‐distearoyl‐*sn*‐glycero‐3‐phosphocholine (DSPC)—with patented differences in both exact composition and ratios of these lipids. In both cases, the ionizable lipid is what is thought to produce an adjuvant response. When increasing molar ratios of ionizable lipids in the LNP formulation, the induction of proinflammatory cytokines such as IL‐6 was found to subsequently increase.^54^ Additionally, the ionizable lipid directly impacts the humoral responses generated to the vaccine. When LNPs were co‐administered with antigen proteins, antigen‐specific antibodies were generated regardless of the inclusion or exclusion of encapsulated IVT‐mRNA.^54^ However, in the case where LNPs lacked ionizable lipids when injecting antigen proteins, antigen‐specific antibody could not be produced. This suggests that ionizable lipids play a crucial role in both antigen generation and innate immune signaling, although the precise mechanism responsible for immune activation remains incompletely understood. The PEGylated lipids in the formulation protect the nanoparticle from opsonization and phagocytosis, allowing for prolonged circulation.^56^, ^57^ Lastly the helper lipids, cholesterol and DSPC, enhance the stability of the nanoparticle by stabilizing the antigen encapsulation and enhancing membrane rigidity which leads to overall greater efficacy and biodistribution.^57^, ^58^, ^59^ Once the host cell is infected, the mRNA is translated into a SARS‐CoV‐2 spike protein that expresses on the host cell. The vaccines, although similar, have different storage ability. Pfizer's BNT162b2 needs to be stored at −60°C to −80°C and can be stored for 6 months, while Moderna's mRNA‐1273 vaccine can be stored at 2°C–8°C for up to 30 days.^59^

#### 3.2.2 Matrix‐M

Matrix‐M is a 40 nm spherical saponin‐based nanoparticle that is a mixture of two purified fractions from the bark of the Quillaja saponaria tree, Fraction‐A and Fraction‐C, formulated with cholesterol and phospholipids.^60^ There are two saponin fractions, Matrix‐A and Matrix‐C, that make up Matrix‐M in combination.^38^ In the clinic, Matrix‐M nanoparticles make up 20.3% of unique vaccine formulations and ~25% of total trials among the nanoparticle category (Figure 3a) and are currently being used for vaccination against a variety of diseases including Epstein–Barr Virus infection, Malaria, and COVID‐19. In a recent study that tested the immunostimulatory properties of Matrix‐M without antigen in mice, the adjuvant was found to increase the number of immune cells in the lymph nodes and spleen by 3‐fold and to induce a strong Th1 response.^60^ At the injection site, Matrix‐M enhances infiltration of innate immune cells such as neutrophils, monocytes, macrophages, natural killer cells, and dendritic cells and promotes the release of proinflammatory cytokines. This cytokine release initiates the influx of more innate immune cells instigating a sequential local immune response that unfolds over the subsequent 24–48 h. Matrix‐M, along with the antigen, also drains to the lymph nodes, leading to increased B‐cell activation and proliferation, along with the activation and polarization of T‐cells. B‐cells further proliferate and differentiate into memory B cells and plasma cells, producing high‐affinity antibodies that protect against the targeted infection threat by the vaccine.^61^ Saponin‐based adjuvants have been known to be advantageous and have been used for animal vaccines in the past as well as in liposome formulation QS‐21 in the adjuvant AS01b, utilized in a licensed vaccine.^62^ As mentioned earlier, Matrix‐M is a combination of Matrix‐A and Matrix‐C saponin fractions, while QS‐21 contains Matrix‐C. Due to its instability, QS‐21 is formulated with cholesterol in liposomal‐based adjuvants like AS01_B_.

#### 3.2.3 Betulin‐based spherical nanoparticles

Betulin‐based spherical nanoparticles are 100–180 nm in size. Betulin is formulated from pentacylic lupade‐type terpenoids produced from the bark of birch trees and has shown to be antifungal, antiviral, and anticarcinogenic.^63^, ^64^ Currently in the clinical landscape, betulin‐based nanoparticles appear in one trial for a COVID‐19 vaccine called Betuvax‐CoV‐2 (NCT05270954). Betuvax‐CoV‐2 contains recombinant RBD‐SD1‐Fc fusion proteins on the surface of a betulin‐based spherical nanoparticles, which is formulated to mimic the SARS‐CoV‐2 virus to trigger the immune system similarly.^65^, ^66^ In recent studies on Betuvax‐coV02, it demonstrated to be safe and tolerated with mild reactions to the vaccination. There were shown to be specific IgG antibody titers and a strong CD4+ and CD8+ T‐cell responses to the antigen.^65^ The mechanism of immunomodulation of betulin is limited, but betulin has been demonstrated to enhance the proliferation of human peripheral blood lymphocytes, as well as enhance anti‐inflammatory and pro‐inflammatory activity in macrophages.^64^, ^67^ Due to betulin being an extraction from a common natural resource, the abundance and low cytotoxicity of the compound makes it even more favorable as vaccine adjuvants.

#### 3.2.4 Gold nanoparticles

Gold nanoparticles (GNP) are specifically used in vaccines to increase antigen stability, accumulation of antigen in lymph nodes, and effective antigen uptake by antigen‐presenting cells.^68^ In the current clinical study landscape, there is one trial in which gold nanoparticles are utilized, specifically for COVID‐19, in a biologic called PepGNP‐COVID‐19, a transdermal COVID‐19 vaccine (NCT05633446). While it is unclear how this precise formulation binds the COVID‐19 antigen, GNPs can be bound to antigens in several ways such as chemical conjugations by thiol and amine linkage, adsorption, and encapsulation.^69^, ^70^ The size of GNPs is crucial for the immune response. When comparing a spherical 20 and 40 nm GNP, the larger spherical GNP was found to produce cytokines such as IL‐6 and IL‐12 more efficiently.^71^ The shape of the GNP is also important. When bone marrow‐derived dendritic cells were treated with rode‐like GNPs, there was an enhanced production of two specific pro‐inflammatory cytokines including IL‐1b and IL‐18.^72^ In comparison, cubical and spherical GNPs, like the ones seen in the clinical vaccine, led to elevated levels of different pro‐inflammatory cytokines such as tumor necrosis factor‐alpha (TNFa), IL‐6, IL‐17, and granulocyte‐macrophage colony‐stimulating factors.^71^, ^72^

#### 3.2.5 Nanostructured lipid carriers

Nanostructured lipid carriers (NLC) are known as the second‐generation lipid nanoparticle that can potentially overcome limitations of previous lipid nanoparticles. They are comprised of a lipid matrix core composed with both liquid and solid lipids, forming a crystallized structure, enveloped by lipidic and surfactant shells, as opposed to LNPs with a solid lipid core. Specifically, NLCs exhibit higher drug loading capacity due to their imperfect crystal structure, prevent drug expulsion by avoiding lipid crystallization and increase drug solubility and controlled release.^73^ Currently, there is one active trial in which a self‐amplifying RNA (saRNA) antigen is bound to the exterior of the NLC. This intranasal vaccine, AAHI‐SC2, was developed by Access to Advanced Health Institutes (AAHI) for the protection against COVID‐19 (NCT05370040). The NLC utilized in this vaccine measures around 125 nm with a core comprised of solid lipids, trimyristin, and liquid lipids (squalene) and a shell comprising surfactants and a cationic lipid, 1,2‐dioleoyl‐3‐trimethylammonium‐propane (DOTAP), which adds in complexing the RNA.^74^ The platform makes for a great candidate for global distribution as it allows for long‐term stability when refrigerated as a liquid while maintaining particle size and concentration for at least a year.^75^ The vaccine can be easily formulated from its liquid form by mixing the vaccine RNA with the NLC to create a NLC/RNA complex that forms from electrostatic interactions with the cationic lipid, DOTAP.^75^ Additionally, the entire complex can be readily lyophilized and stored at room temperature for 8 months or refrigerated for nearly two years.^76^ This thermostability is an improvement on what is seen with previous LNP formulations and could significantly improve distribution of vaccines. The AAHI‐SC2 vaccine was demonstrated to produce a high serum‐specific IgG and SARS‐CoV‐2 Wuhan‐strain specific neutralizing antibodies.^77^ When comparing intramuscular prime and intranasal booster in mice, there was a robust increase in IFNγ‐secreting T‐cells with a strong Th1‐biased response and negligible Th2 and Th17 responses. In addition, there was robust respiratory mucosal immune response with SARS‐CoV‐2‐reactive lung‐resident memory and lung‐homing T cell populations for the intranasal route. The heterologous administration method with an intramuscular prime and intranasal boosting route demonstrates potential to maximize a robust mucosal and systemic immunity with this adjuvant.

#### 3.2.6 Lipid inorganic nanoparticles

Lipid inorganic nanoparticles (LION) are 52 nm particles designed to enhance the stability, delivery, and immunogenicity of therapeutics. In the current clinical trials, LIONs are utilized in two unique vaccine formulation across three total trials focusing on vaccination against COVID‐19. In both formulations, LION is combined with self‐replicating mRNA (repRNA) encoding for the spike protein of SARS‐CoV‐2 bound to the outside of the LION, resulting in the vaccine under the name QTP104 in South Korea developed by Quartis (NCT05876364) and HDT‐301 in Brazil and the US developed by HDT Bio (NCT05132907, NCT05542693). LION is composed of a squalene emulsion core with 15 nm superparamagnetic iron oxide (SPIO) nanoparticles embedded in this phase for enhanced stability and surrounded by surfactants and the cationic lipid DOTAP.^78^ Squalene‐based adjuvants are well known as mentioned throughout this review, while SPIO nanoparticles have history in the clinic in MRI contrast and iron replacement therapies. Once the LION is formulated, it is combined with the RNA resulting in a LION complex. The complexation of the RNA is like that seen with NLCs in which the cationic lipid, DOTAP, forms electrostatic connections with embedded RNA molecules. An advantage of LION is that it can be stable for at least 3 months at room temperature, making it an excellent candidate for global distribution. Ongoing studies on repRNA‐CoV2S, an RNA replicon in LION formulation, have shown to induce antigen specific T‐cell and antibody responses for the SARS‐CoV‐2 spike protein. Additionally, there was a largely Th1 biased immune response with the vaccine inducing both anti‐spike protein IgG antibodies and a robust T‐cell response, with higher dosages enhancing the Th1 responses.^79^ A single intramuscular immunization in mice created a 100% seroconversion after 14 days as well as a robust antigen‐specific IgG response. The immunization was also compared between younger and older mice and mice across all ages had a robust splenic T‐cell response with variable T‐cell responses in the oldest 17‐month‐old group. Non‐human primates such as macaques showed consistent results with mice as well and indicated a long term antigen‐specific memory T‐cell response.^78^

### 3.3 Viral vectors

Viral vectors are modified viruses used universally to deliver target genes to host cells. In vaccination they have been largely identified as antigen carriers, delivering DNA antigen as well as protein antigen to immune cells. However, viral vectors also have adjuvant capabilities as they can imitate natural infections caused by viruses. Generally, a natural infection occurs through viral attachment to the host cell through a surface receptor. This presence of the virus triggers the innate immune responses effectively through the recognition of pathogenic structures or PAMPs on the vector, as well as interaction with pattern recognition receptors (PRR).^80^, ^81^, ^82^ Although the vectors induce a viral‐like infection, the viral vectors are modified to be non‐virulent and replication‐deficient to avoid further virus‐specific infection.^81^ While potent adjuvants, viral vectors have a few drawbacks. Largely, it is difficult to modulate the innate response initiated and at times vectors can cause too intense of an innate reaction leading to cytotoxicity, immunity against repeated doses, and robust inflammatory responses that can reduce the effectiveness or even get cleared by the body.^83^, ^84^

**FIGURE 4:**
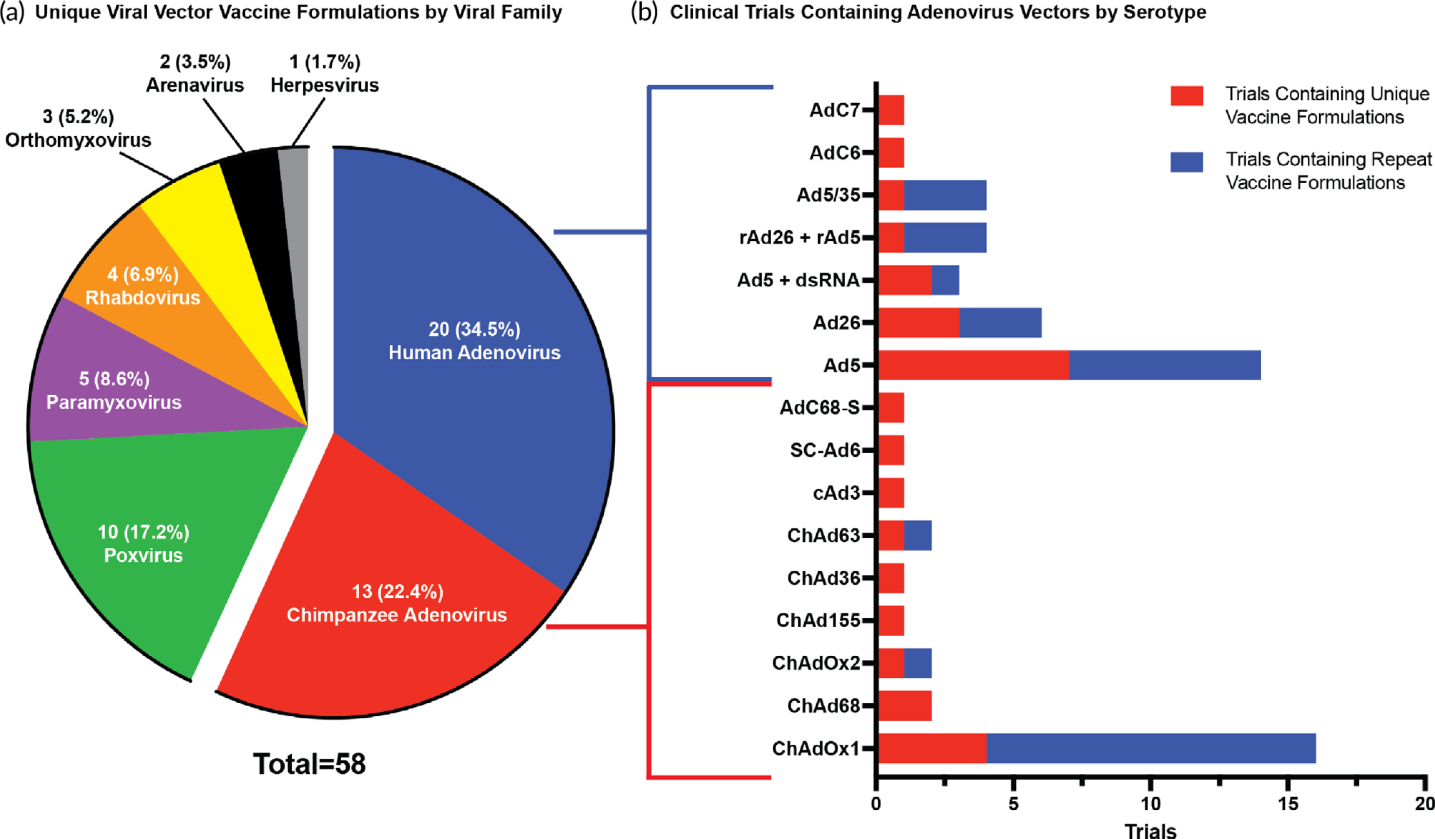
Viral vectors in the clinic. (a) Make‐up of unique vaccine formulations containing viral vectors in the clinical trial space separated by viral family. The number of unique vaccine formulations as well as the resulting percent of the whole space is provided. (b) The number of clinical trials that contain an adenovirus vector by serotype. This is defined by both number of trials with unique vaccine formulations and number of trials with repeat formulations.

Vectors utilized in the clinic are derived from several virus families including Paramyxovirus, Adenoviridae, and Orthomyxoviridae. Within each family, there are several different viral vectors that are modified through gene editing to avoid preexisting immunity, as well as reduce toxicity and immunogenicity.^85^ As mentioned earlier, there is one viral vector adjuvant, rVSV, that has been utilized in the licensed rVSV‐ZEBOV Zaire Ebola vaccine by Merck. There have also been two viral vectors granted temporary emergency approval by WHO and the FDA. The ChAdOx1 vector in AstraZeneca's COVID‐19 vaccine was granted emergency approval by WHO in February 2021, but this approval has since been removed. Additionally, the Ad26 vector utilized in Janssen's COVID‐19 vaccine received emergency approval in February of 2021 by the FDA, but this approval has been removed for to the potential risk of blood clots associated with the vaccination. In the current clinical landscape, there are 27 viral vector adjuvants ranging across eight viral families (Figure 4a). Among the adjuvants in clinical trials, 15.8% of total trials and 13.6% of unique vaccine formulations utilize viral vectors, making it the third largest group of adjuvants in the clinical trial vaccines landscape (Figure 2).

#### 3.3.1 Rhabdovirus

rVSV is the only viral vector in the Rhabdovirus family being tested in clinical trials. rVSV is a live, attenuated recombinant vesicular stomatitis (Indiana) virus (VSV).^86^ This vector is currently evaluated in four unique vaccine formulations across five total trials in vaccines against Nipah Virus Infection, Lassa Virus infection, and, Ebola Sudam Virus Disease (ESVD). VSV is a 11‐kb non‐segmented, single‐stranded, negative‐sense RNA virus composed of 11,000–12,000 nucleotides that encode for five genes: N, P, M, G, and, L.^87^ The virus primarily infects insects, horses, cattle, and swine, therefore avoiding pre‐existing immunity in humans and increasing the efficacy of VSV viral vector vaccines.^88^ VSV's glycoprotein G, a class III viral fusion protein, allows for cell attachment and membrane fusion and enables infection.^89^ In the licensed rVSV ZEBOV vaccine, VSV‐EBOV, the EBOV glycoprotein (GP) is integrated into a VSV vector, in which the G open reading frame is intentionally removed. This modification allows for the replication of rVSV displaying the morphology of a rhabdovirus while expressing EBOV GP on its surface.^90^ Like other viral vectors, rVSV generates signals that interact with the host's Toll‐like receptors (TLRs) and pattern recognition receptors. This interaction triggers the innate immune response, consequently boosting the adaptive immune response.^84^ The VSV viral vector has shown to be a promising platform due to its serovalence, high replicability, and low to mild symptoms in vaccinated individuals. However, while effective in producing an immune response to the desired antigen, the licensed vaccine also demonstrated cytotoxic T‐cell and antibody responses against the carrier itself, VSV, in more than one‐third of the subjects; this can directly affect the replication abilities of VSV‐EBOV.^91^ Despite this, this carrier continues to be explored to various vaccines due to its positive impacts on immune response and high manufacturability: rVSV can be produced at high titers through transfection of mammalian cells or HEK293T expressing the gene of interest in replacement for the G‐protein.^92^

#### 3.3.2 Adenoviruses

Adenoviruses are non‐enveloped double‐stranded DNA icosahedral‐shaped viruses and dominate the viral vector space with both human and chimpanzee adenovirus vectors.^93^ Among the Adenoviridae family, the genus *Mastadenovirus* consists of serotypes that infect humans and nonhuman primates such as bovine, canine, and simian species such as chimpanzees.^94^ The serotypes are further divided into species A‐G. In the current clinical trial space, human adenovirus vectors represent the largest viral vector family evaluated with 17 unique vaccine formulations across 33 trials investigating many COVID‐19 vaccines, along with HIV, Rabies, Cholera, and others (Figure 4a). Among the 56 human adenovirus serotypes, wild type serotypes cause mild to moderate symptoms of respiratory or gastrointestinal diseases.^95^ These diverse strains often result from natural genetic variations that occur during viral replication. The numerical designations help scientists classify and study these variants, with each number representing a distinct genomic and serotypic identity within the extensive family of human adenoviruses. Among the clinical trial landscape, Ad5 is the most used viral vector, consisting of 10 unique vaccine formulations for protection against COVID‐19 and Norovirus (Figure 4b). Over the course of the COVID‐19 pandemic, Ad5 viral vectors have been used as a COVID‐19 vaccine platform with several vector modifications for COVID‐19 vaccines being tested in clinical trials. In a recent study, it was shown that varying dosage did not significantly increase the levels of antibody production, demonstrating the limits of adenoviral vectors.^96^ Ad5 viral vectors were demonstrated to produce specific antibodies and long‐term T‐cell response but proved high seroprevalence as 52% of participants had high pre‐existing immunity.^97^ Ad5 vector has been demonstrated to be effective and safe, but due to its high seroprevalence, many other derivations have been created from rare human adenovirus species and non‐human primates that appear in the clinical trial space today such as Ad26 for COVID‐19 vaccine called Ad26.COV2.S (NCT05515042) (Figure 4b).^98^, ^99^

As Chimpanzee adenoviruses (ChAd) are phylogenetically akin to human adenoviruses, there are many similar traits between the two families of adenoviruses. ChAds are found in 13 unique vaccine formulations across 27 total trials in the clinical trial landscape, making up the second largest viral vector group (Figure 4a). ChAd vectors are in trials for various infectious diseases including HIV, COVID‐19, Rabies, and Ebola. Among the ChAd vectors, ChAdOx1 is the most prevalent in clinic with four unique vaccine formulations across 16 total trials (Figure 4b). ChAd viral vectors are simian adenoviruses that originate in the genus Mastadenovirus of the Adenoviridae family; they are mainly structured as non‐enveloped double‐stranded DNA and are capable of infecting human and nonhuman primates.^100^ The most used simian adenoviruses vector, ChAdOx1, is derived from chimpanzee adenovirus Y25 and developed by Oxford University. This vector is safe and highly manufacturable, and has a storage capacity at 2°C–8°C, making it ideal for global distribution.^98^ ChAdOx1 also avoids pre‐existing immunity in human adenoviruses due to its chimpanzee origin. In a study done on a single dose ChadOX1 nCoV‐19 (AZD1222), it was found to induce a strong Th1‐immune response, IgG1 and IgG3 antibody production, as well as populations of CD8 T‐cells, natural killer cells, and B cells.^101^ Like ChadOX1, many of the other vectors such as ChAd155 and ChAd36 show similar immune responses with a robust humoral and cellular response.^92^, ^101^, ^102^ Overall, ChAd vectors are effective and ideal for global distribution, while avoiding pre‐existing immunity found with human adenovirus vectors.

#### 3.3.3 Poxviridae viruses

Modified Vaccinia Ankara (MVA) is the only viral vector in the Poxviridae family and is used in 10 unique vaccine formulations across 11 total trials in the clinic, making up 17.2% of unique vaccine formulations in the viral vector landscape (Figure 4a). Poxviruses are double‐stranded DNA viruses that replicate in the cytoplasm of host cells, thus eliminating the integration of the virus in the host's genome. Usually known to infect humans and animals, poxviruses typically causing lesions, rash, and skin nodules.^103^ The MVA vector is a highly attenuated strain of vaccinia virus and has shown to be safe among humans including individuals with HIV infection, while being a versatile platform that can be stored long term. Lyophilized MVA vaccines were proven to be stored at 37°C up to 2 weeks and longer than a year when refriegerated.^104^ MVA is largely being tested in clinical trials for upper respiratory diseases such as COVID‐19, Respiratory Syncytial Virus, Middle East Respiratory Syndrome (MERS), and malaria. It has demonstrated to induce high numbers of antigen specific CD8 and CD4 T‐cell responses and fast memory differentiation.^105^ In a recent study, the administration of MVA‐EBOV‐GP, a viral vector vaccine against the Ebola virus (EBOV) glycoprotein (GP), led to the generation of virus‐neutralizing antibodies, while MVA‐EBOV‐NP, a viral vector vaccine against the Ebola nucleoprotein (NP), triggered the development of cytotoxic CD8 T‐cells specific to NP. Both the neutralizing antibodies and the NP‐specific CD8 T‐cells demonstrated equal effectiveness in eliciting a protective immune response.^104^

#### 3.3.4 Paramyxovirus

Two viral vectors from the paramyxovirus family are currently being tested in clinical trials: PIV5 and Newcastle Disease Virus (NDV), representing five unique vaccine formulations across nine total trials (Figure 4a). Paramyxovirus are enveloped virions sized 150–300 nm. They are single‐stranded RNA viruses that replicate in the cytoplasm of the host, like the Poxviridae family. The virion contains two glycoproteins: the trimeric fusion (F) protein enables membrane fusion and glycoproteins hemagglutinin‐neuraminidase (HN), hemagglutinin (HA), or glycoprotein (G) enable viral attachment.^106^ These viruses are well known for infecting vertebrates for infections such as mumps virus, measles virus, respiratory syncytial virus (RSV), and parainfluenza virus.^107^, ^108^ Both viral vectors, PIV5 and NDV contain the same two transmembrane glycoproteins: the hemagglutinin‐neuraminidase protein, HN, and fusion protein F.^108^, ^109^ These vectors are currently in trials for upper‐respiratory infections such as COVID‐19 and Syncytial Virus. PIV5 demonstrates low virulence in mammals, except for upper respiratory infections in dogs, and is highly manufacturable as it can be grown in Vero cells up to 8 × 10^8^ PFU/ml.^109^ In a recent study for an intranasal PIV5 RSV vaccine, approximately 50% of participants were seropositive at baseline for PIV5 neutralizing antibodies but demonstrated to have robust cellular immune responses against RSV F protein; thus, the prior exposure to this viral vector does not quench the response.^110^ Similarly, NDV can be mass‐produced at low costs, as it is an avian virus that can be grown in embryonated chicken eggs.^111^ NDV has no preexisting immunity among humans but is known to be contagious and dangerous among avian species. In the past, NDV has been used as a safe candidate for cancer therapies and has demonstrated to have oncolytic advantages through induction of type I and III antiviral interferon in tumor cells and type III for noncancerous cell.^112^, ^113^

#### 3.3.5 Orthomyxoviridae viruses

The orthomyxoviridae family consists of spherical‐like shaped viruses composed of segmented, single‐stranded negative‐strand RNA. This family most notably encompasses influenza viruses which are represented in the current clinical trial viral vector space. There are three unique vaccine formulations across two trials containing variations of the influenza A virus (Figure 4a), specifically H1N1 and H3N2, utilized in vaccines against COVID‐19. Influenza viruses can infect mammals and birds, causing mild to severe respiratory diseases in humans and have four subtypes: A, B, C, and D. The subtypes are created based on proteins hemagglutinin(H–H18) and Neuraminidase (N1‐N11), with common subtypes H1N1 and H3N2 being the common virus to circulate among humans.^114^ Hemagglutinin (HA) is crucial for viral entry into the host, while neuraminidase (NA) facilitates the release of newly formed viral particles from infected cells. One of the vaccines being evaluated in clinic, Corfluvec, utilizes two different attenuated influenza vectors to be delivered intranasal with recombinant H3N2 vector utilized in the prime and recombinant H1N1pdm09 vector based off the H1N1 virus that appeared in 2009 used in the boost (NCT05696067). DelNS1‐2019‐nCoV‐RBD‐OPT1 is also being evaluated in clinic for protection against COVID‐19; this vaccine is delivered intranasal utilizing an influenza A H1N1 vector (A/California/4/2009, CA4) (NCT05200741). While in both trials the vector utilized is a weakened strain, the impact of pre‐existing immunogenicity to the vectors on the overall efficacy and reactogenicity of the vaccines are unclear and something to be further elucidated.

#### 3.3.6 Arenaviruses

The arenaviruses family consists of enveloped single‐stranded viral RNA. From this family the pichinde virus (PICV) and lymphocytic choriomeningitis virus (LCMV) make up two unique vaccine formulations delivered in combination with an alternating‐vector immunization strategy testing a Chronic Hepatitis B therapeutic vaccine (NCT05770895). PICV is a nonpathogenic RNA virus that was derived from *Oryzomys albigularis*, also known as rice rats.^115^ It has shown low seroprevalence in the human population in areas with infected rodents which mitigates pre‐existing immunity in PICV vector vaccines.^116^ LCMV consists of bisegmented negative‐stranded RNA, but unlike PICV, LCMV is a pathogenic virus causing several neurological diseases such as meningitis, encephalitis, and birth defects in infants through virulent rodents.^117^ For safety, LCMVs undergo genetic mutations that attenuate replication and reduce pathogenicity in vaccines.^118^ The Arenavirdae family has been utilized in past immunological research due to their abilities to induce robust and functional cytotoxic T‐cell responses; however, the use of these two genealogically related vectors in a prime‐boost combination enhances this response and avoids vector‐neutralizing antibodies.^119^

#### 3.3.7 Herpesviridae viruses

Herpesviridae family consists of a large double‐stranded DNA genome. Human cytomegalovirus (HCMV) is the only viral vector from the herpesviridae family currently in clinical trials, with one trial for an HIV vaccine, VIR‐1388, developed by Vir Biotechnology (NCT05854381). This vaccine has shown to produce HIV‐specific T cells using a weakened CMV virus containing HIV material for protection against chronic HIV infection. HCMV has demonstrated prevalence in 80% of the population in Europe and the United States and 100% in Africa and Asia.^120^ HCMV infections are typically asymptomatic in healthy individuals but can pose severe health risks in patients with compromised immune systems, including infants and those with AIDS. Symptoms may manifest as CMV mononucleosis, fever, leukopenia, pneumonitis, and gastrointestinal diseases; and in patients with AIDs can often cause retinitis.^121^ However, this vaccine is currently utilized in adults of good health without HIV, and due to HCMV's ability to be asymptomatic in most adults, it is a good candidate as a viral vector in healthy individuals. Additionally, in a study on murine CMV (MCMV), the virus was shown to induce T‐cell responses even in seropositive patients; while this trait makes it difficult to protect against CMV it can be exploited in use as a vector.^122^ HCMV is a special case as it produces a high frequency of virus specific CD4+ T cells with a range of cytotoxic and effector functions, as well as CD8+ T cell population acting as effector memory cells throughout the lifetime of the host. This phenomenon is known as “memory inflation,” making it an effective viral vector with potential for long‐term immunity, especially for diseases such as HIV which require strong cellular responses.^123^ In non‐human models, CMV vector vaccines also demonstrated the ability to be genetically altered and program diverse CD8+ T cells with varying epitope targets. This allows CMV vectors to be tailored for specific CD8+ T cell responses to target varying disease models.^124^

### 3.4 TLR agonists

TLRs are a type of pattern recognition receptor (PRR) which are innate immune receptors with the ability to recognize pathogen‐associated molecular patterns (PAMPs) from microbes or danger‐associated molecular patterns (DAMPs) from damaged tissue. PRRs were first proposed by Charles Janeway in 1989 when he hypothesized they served as the connection between the innate and adaptive immune system.^125^ Following this prediction, TLR4 was first discovered in 1996, and in 1998 it was found that LPS can bind to this receptor. Since then, other TLR receptors have been identified with unique innate immune pathways initiated. TLR agonists were first utilized in the AS04 adjuvant in the HPV vaccine licensed by the FDA in 2009. AS04 contains a TLR4 agonist, MPL that is also utilized in the adjuvant AS01b that was utilized in the zoster vaccine, licensed for use in 2017. With both AS04 and AS01b, MPL is absorbed to another immune modulator such as aluminum or the liposomal formulation of QS‐21. Two other TLR agonists (CpG 1018 and CpG 7909) have been utilized in licensed vaccines for use in clinic in 2017 and 2023, respectively, each being unmethylated CpG motifs which act on TLR9.

**FIGURE 5:**
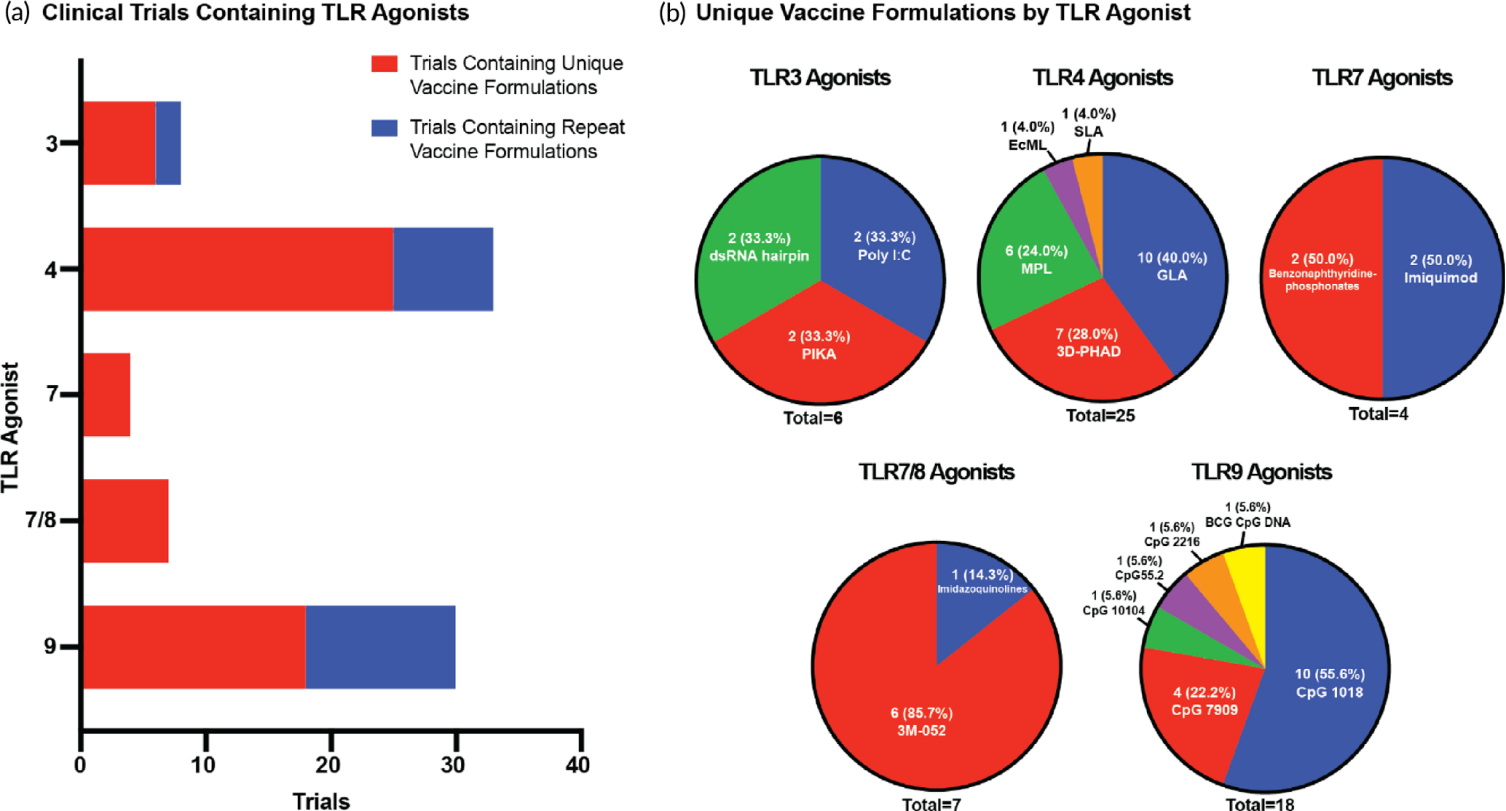
TLR Agonists in the clinic. (a) The number of clinical trials that contain a TLR agonist by TLR number. This is defined by both number of trials with unique vaccine formulations and number of trials with repeat formulations. (b) Make‐up of unique vaccine formulations containing a TLR agonist in the clinical trial space divided by TLR agonist type and further specified by the agonist utilized. The number of unique vaccine formulations as well as the resulting percent of the whole space is provided.

In the current clinical trial landscape, 60 unique vaccine formulations contain a TLR agonist representing about 15% of all novel vaccine formulation being evaluated (Figure 2). There are five TLRs targeted currently in the clinic trials—TLR3, 4, 7, 7/8, and 9—with multiple agonists being evaluated to initiate these pathways (Figure 5a, b). TLR4 agonists have the largest presence in the identified clinic trials, appear in about 42% of all unique vaccine formulations that contain a TLR agonist. Two TLR agonists specifically dominate the current clinical space. CpG 1018, a previously utilized adjuvant in licensed vaccines and TLR9 agonist, and GLA, a novel TLR4 agonist, each represent about 17% of novel vaccine formulations containing a TLR agonist. Despite the history of TLR4 and TLR9 agonists, and their resulting dominance in the field, each TLR pathway prompts its own unique immune cascade that can be important in harnessing a complete response to the pathogen. Therefore, the presence of other TLR agonists in active clinical trials is promising in expanding this class of adjuvants.

#### 3.4.1 TLR3 agonists

TLR3 agonists represent a small group of materials currently utilized in clinic with only six unique vaccine formulations being evaluated across eight total trials (Figure 5b). Among immune cells, TLR3 is specifically found on myeloid dendritic cells (mDCs), macrophages, and mast cells, primarily localized in the endosomes of these cells. Upon binding of the ligand, TLR3 associates with the adaptor protein TRIF leading to an anti‐viral response marked by an increase in type I interferons (IFN) secretion.^126^ The activation of TLR3 on DCs is incredibly important as this initiates a potent IFN release and induces antigen‐specific immune responses important for the adaptive immunity.^127^ The strong IFN response and innate immune cascade resulting from TLR3 activation has made this pathway important for protection against numerous viruses such as influenza,^128^ RSV,^129^ among others.^130^, ^131^ This receptor recognizes double‐stranded RNA (dsRNA) which is a viral replication intermediate and therefore mimicked by the TLR3 agonists in clinic.

There are three TLR3 agonists each being explored in two unique vaccine formulations in the identified clinical trials—Poly I:C, PIKA, and a dsRNA hairpin—with only PIKA being utilized as the only adjuvant in its formulation (Figure 5b). Polyinosinic: polycytidylic acid (Poly I:C) is a synthetic dsRNA analog that is a common TLR3 agonist utilized in both cancer and infectious disease applications. In clinic, Poly I:C is currently utilized in two distinct vaccine formulations across three trials (Figure 5b). It is combined with QS‐21 and lipid molecules to create MA105 which is being tested as an adjuvant in the herpes zoster vaccine (NCT05856084). Additionally, it is combined with two other components, a host‐defense peptide IDR‐1002, and a cationic polymer adjuvant, polyphosphazene (PCEP), to create TriAdj which is being investigated for a novel COVID‐19 vaccine (NCT05693272). The other TLR3 agonist used in combination is the dsRNA hairpin that is formulated with Ad5 viral vector for use in an oral norovirus (NCT05626803) and oral COVID‐19 (NCT05067933) vaccine both developed by Vaxart. Utilizing a TLR3 agonist for this administration route allows for a more robust innate immune response in the gut since TLR3 can be found on intestinal epithelial cells.^132^ In this system the dsRNA hairpin is incorporated into the viral vector and encoded on the same viral vector as the antigen. When evaluated with an influenza model in mice, the inclusion of the dsRNA hairpin in the vector was shown to enhance the antibody response to the antigen.^133^ The final TLR3 agonist evaluated in the identified trials for both a rabies (NCT05667974) and COVID‐19 (NCT05463419) vaccine is PIKA, a stabilized chemical analog of dsRNA developed by YS Biopharma. In both formulations, PIKA interacts with TLR3 and initiates the innate immune pathway associated with this receptor.

#### 3.4.2 TLR4 agonists

TLR4 agonists are the most prevalent TLR agonists currently evaluated in active clinical trials (Figure 5a). This is likely due to the FDA approval history of the TLR4 agonist MPL which is present in AS04 and AS01B. TLR4 is a transmembrane protein expressed in immune cells mainly of the myeloid origin, including monocytes, macrophages, and dendritic cells. It is also found on non‐immune cells such as epithelial, neurons, and cells of the central nervous system (CNS).^134^ TLR4 was first discovered through its ability to sense LPS, however, it also is capable of binding endogenous molecules produced by tissue injury known as DAMPs. Importantly, TLR4 alone cannot sense LPS. Instead, TLR4 must be physically associated with MD‐2 on the cell surface to respond to LPS, forming a LPS/TLR4/MD‐2 complex to initiate the resulting immune pathway in the cell.^135^ Once initiated TLR4 can trigger two separate and competitive pathways: MyD88‐independent (TRIF dependent) and MyD88‐dependent pathways.^136^ The association of adaptor proteins, such as MyD88, to the TLR4 complex dictates the resulting immune response. The MyD88‐dependent response results in the release of proinflammatory cytokines such as IL‐6 and TNF‐a while the MyD88‐independent response leads to type 1 IFN release.

As discussed in the licensed vaccine adjuvant section, MPL is a non‐toxic derivative of LPS derived from *Salmonella minnesota* R595 containing multiple congeners of monophosphoryl lipid A (hexa‐acylated, penta‐acylated, tetra‐acylated, and tri‐acylated) and is utilized in combination with both aluminum salts and QS‐21 in previously licensed adjuvant systems to target the TLR4 pathway. Lipid A is a conserved molecular pattern of LPS, and the main inducer of immunological responses to LPS, making it a key focus for use as an adjuvant.^137^ Clinical grade MPL is manufactured exclusively by GSK for use in their adjuvant systems, while other “generic” forms of monophosphoyl lipid A are also used in clinic and are designated as MPLA. For this review both MPL and MPLA will be referred to as MPL. MPL has been shown to activate both the MyD88‐dependent and TRIF‐dependent pathways but biases towards the TRIF‐dependent pathway, which aids in its low toxicity compared to LPS and its overall adjuvant effects.^138^ Due to its natural derivation, MPL has some inherent heterogeneity and manufacturing hurdles, prompting the generation of a pure synthetic hexa‐acylated lipid A derivative, glucopyranosyl lipid A (GLA).^139^, ^140^ MPL and other naturally derived endotoxins can have a variety of number of chains, attachment sites, and carbons within the chains; whereas GLA, as a synthetic alternative, has a defined number and lengths of chain attachments and carbons.^140^ GLA produces a similar response to MPL with reduced endotoxicity compared to LPS, a bias towards signaling through the TRIF‐dependent pathway, and similar dose and time‐dependent responses in mouse dendritic cells. However, it has shown stronger stimulatory abilities on human monocyte‐derived dendritic cells.^140^ Another synthetic MPL, 3D‐PHAD, is also prevalent in the current clinical trial space and found in the ALF family of adjuvants—ALFQ and ALF43. While GLA is the synthetic structure analog to hexa‐acylated MPL, 3D‐PHAD is a pure penta‐acylated synthetic analog to 3‐deacylated monophosphoryl lipid A derived from bacterial LPS and has shown equivalency to GLA in pre‐clinical analysis. GLA, 3D‐PHAD, and MPL have a notable presence in the current clinical trial space with GLA being the most present in about 17% of vaccine formulations with TLR agonists, 3D‐PHAD representing about 12%, and MPL representing 10% (Figure 5b). Each of these agonists are formulated in combination with other adjuvants or carriers, with only one vaccine formulation being evaluated with just the TLR agonist, MPL (NCT04066881). When used in combination with other materials, these TLR4 agonists are commonly formulated with alum (AS04), QS‐21 liposomes (AS01b, AS01e, ALFQ), or in the case of GLA a squalene emulsion (GLA‐SE). MPL, GLA, and 3D‐PHAD are being evaluated in active clinical trials in a variety of vaccines protecting against parasitic and bacterial infections such as schistosomiasis and leprosy to viral infections such as HIV and COVID‐19.

While MPL, GLA, and 3D‐PHAD dominate the TLR4 clinical trial space, two other TLR4 agonists are currently being evaluated. Second‐generation lipid adjuvant (SLA) was developed by the same company to develop GLA—AAHI—and was designed based off structural knowledge of the TLR4/MD‐2 complex as opposed to MPL. SLA was designed to better fit into MD‐2 by removing carbons from the end of GLA's acyl chains, which allows for a more compact interaction with the TLR4/MD‐2 complex. This compact binding results in a shift in TRIF pathway‐bias compared to GLA, leading to an increase in Th1 chemokines and cytokines.^141^ SLA is formulated with a squalene emulsion and utilized in one clinical trial for protection against herpes zoster (NCT05304351). The final TLR4 agonist evaluated in active clinical trials is EcML, present in one vaccine formulation across two trials in a vaccine against COVID‐19 (NCT05572879). EcML is a purified MPL produced from an engineered *Escherichia coli* (*E.coli*) strain by Eubiologics. It has been shown to produce similar immune effects as MPL in mice while reducing manufacturing time and cost that comes with commercially available MPL.^142^, ^143^ The current formulation of EcML being evaluated in clinic is bound to a QS‐21 liposome.

#### 3.4.3 TLR7 agonists

Sole TLR7 agonists are the least prevalent TLR agonists in the clinical trial space (Figure 5a). TLR7 is an endosomal transmembrane protein found primarily on plasmacytoid dendritic cells (pDCs) and B cells.^144^, ^145^ It is also found at low levels on other non‐immune cell types such as keratinocytes and epithelial cells. TLR7 senses guanosine‐ and uridine‐rich single‐stranded RNA (ssRNA), ssRNA viruses, as well as synthetic antiviral nucleoside analogs.^146^, ^147^ When bound, TLR7 signals through MyD88 cytosolic adaptor protein which through the association and activation of other downstream proteins results in inflammasome activation and the release of proinflammatory cytokines to prompt an innate immune response.^148^

Imiquimod is a synthetic imidazoquinoline amine, in which imidazoquinolines represents a drug class of small (<400 Da) synthetic nucleoside analogs and is the only FDA‐approved agonist to an intracellular TLR to date. While it has not yet been utilized in a licensed vaccine as an adjuvant in infectious disease vaccines, it is presently approved for topical treatment of basal cell carcinoma or genital warts. Imiquimod has been shown to bind TLR7 and induce a variety of proinflammatory cytokines locally, leading to a Th1‐biased immune response.^149^ Currently in the vaccine clinical trial space, imiquimod is being utilized as a topical adjuvant in the protection against both influenza and malaria in which both antigens are administered into the intradermal space. One other TLR7 agonist is being evaluated in clinical trials, without previous approval in other spaces. Benzonaphthyridine‐phosphonate is a TLR7 agonist absorbed onto alum to yield the complete adjuvant AS37 developed by Novartis and later acquired by GSK. Benzonaphthyridines represent a novel class of TLR7 agonists that were optimized through medicinal chemistry by in vitro screening and then evaluated in vivo to yield a top candidate that had low systemic exposure with increased localized innate immune responses and long‐term T‐cell and antibody responses.^150^ The resulting TLR7 agonist was chemically modified with phosphonates to allow adsorption onto alum allowing for enhanced retention in the injection site and bolstering the subsequent immune response.^151^ In the clinical trial space, AS37 is being utilized in vaccination against both *Staphylococcus aureus* and Hepatitis B.

#### 3.4.4 TLR7/8 agonists

TLR7/8 agonists have the unique ability of binding to both TLR7 and TLR8 to prompt a more robust innate immune response. TLR8, similar to TLR7, is found on the endosome membrane; however, it is expressed more on myeloid dendritic cells and monocytes as opposed to pDCs as seen with TLR7.^152^ TLR8 signals through the MyD88 pathway but differs from TLR7 in the cytokine profile induced. While TLR7 is largely associated with the production of IFNα, TLR8 is linked with the production of other pro‐inflammatory cytokines such as TNFα and IL‐12.^153^, ^154^ Thus, the ability to activate both receptors is beneficial in a more complete cytokine induction in response to the adjuvant.

There are two TLR7/8 agonists currently being evaluated in active clinical trials (Figure 5b): imidazoquinoline gallamide (IMDG) and 3M‐052. As mentioned previously, imidazoquinolines represent a drug class of nucleoside analogs with the ability to interact with TLR7 and TLR8 specifically. There is one trial of a vaccine developed by Ocugen for the protection against COVID‐19 adjuvanted with IMDG adsorbed to alum (NCT05258669). IMDG is a dimeric construct of imidazoquinoline molecules linked at the C2 position with the distinct ability to antagonize both TLR7 and TLR8, leading to a Th1 immune response by inducing strong type I interferon responses.^155^ 3M‐052 developed by 3M is the other TLR7/8 agonist with strong prevalence in the clinical trial space. This agonist appears in five clinical trials each evaluating a unique vaccine formulation against HIV and COVID‐19. In all five trials, 3M‐052 is absorbed to alum, with one trial also evaluating the agonist alone without absorption (NCT04915768). 3M‐052 is a synthetic imidazoquinolinone structured similar to resiquimod but with an 18‐C fatty acyl chain which enhances hydrophobicity of the molecule, therefore, reducing systemic diffusion and improving bioavailability in the immunization site and draining lymph nodes.^156^ By activating the TLR7/8 pathways, 3M‐052 induces pro‐inflammatory cytokine release and enhances Th1 immune responses.

#### 3.4.5 TLR9 agonists

TLR9 senses unmethylated CpG dinucleotides which is a hallmark of microbial DNA. Synthetic oligonucleotides (ODNs) containing CpG motifs can mimic this sensing and function as TLR9 agonists. TLR9 is a endosome membrane receptor which is predominately expressed on pDCs and B‐cells among other immune cell subsets^157^ and signals through the MyD88 pathway to initiate type I interferon release for an improved innate immune response to the antigen.^158^ Additionally, TLR9 agonists aid in the biasing of adaptive responses towards Th1 and promote the proliferation of B‐cells.^147^ There are four distinct classes of CpG ODNs with differing activation abilities in human cells. Class B ODNs are the most extensively studied in clinical trials as this includes the two CpG adjuvants, CpG 1018 and CpG 7909, utilized in licensed vaccines. These ODNs contain 1 to 5 CpG motifs embedded in a phosphonothioate (PS) backbone and can trigger pDCs to differentiate and produce TNFα while still stimulating B‐cells to proliferate and secrete IgM.^159^ Class A ODNs contain a single CpG motif which is flanked by palindromic sequences which results in the formation of a stem loop structure. While these ODNs similarly trigger pDC maturation and the release of IFNα, they do not impact B‐cells due to their localization on the early endosome.

Due to its history in licensed vaccines, TLR9 agonists have a strong presence the clinical trial space for TLR agonists with the second most trials behind TLR4 agonists: 30 total trials with 18 unique vaccine formulations (Figure 5a). There are six TLR9 agonists being evaluated in active clinical trials with class B ODNs dominating the space and the previously utilized TLR9 agonist, CpG 1018, as the most prevalent among all TLR9 agonists. CpG 1018 appears in 10 unique vaccine formulations making up 19 total trials for vaccines against COVID‐19, flu, HIV, and plague (Figure 5b). In all but one trial (NCT05506969), CpG 1018 is absorbed to alum. This adsorption has shown to enhance immune responses to the antigen.^160^ CpG 7909, another previously utilized Class B ODN, is the second most prevalent TLR9 agonist utilized in four unique vaccine formulations across five total trials; this agonist is only absorbed to alum in half of its formulations and delivered independently for the rest. Two other class B ODNs are also being evaluated, CpG 10104 and CpG55.2, both utilized in one unique vaccine formulation protecting against Hookworm (NCT03172975) and COVID‐19 (NCT05279456), respectively. While CpG 10104 is being tested adsorbed to alum, CpG55.2 is formulated with a novel polysaccharide adjuvant, Advax, which are delta inulin particles. CpG 2216 is a class A ODN and is being evaluated in one trial for the tuberculosis vaccine, GamTBvac (NCT04975737). CpG 2216 is delivered with a polycationic derivative of dextran, dextran DEAE, for the complete adjuvant system.

#### 3.4.6 Combination materials

**FIGURE 6:**
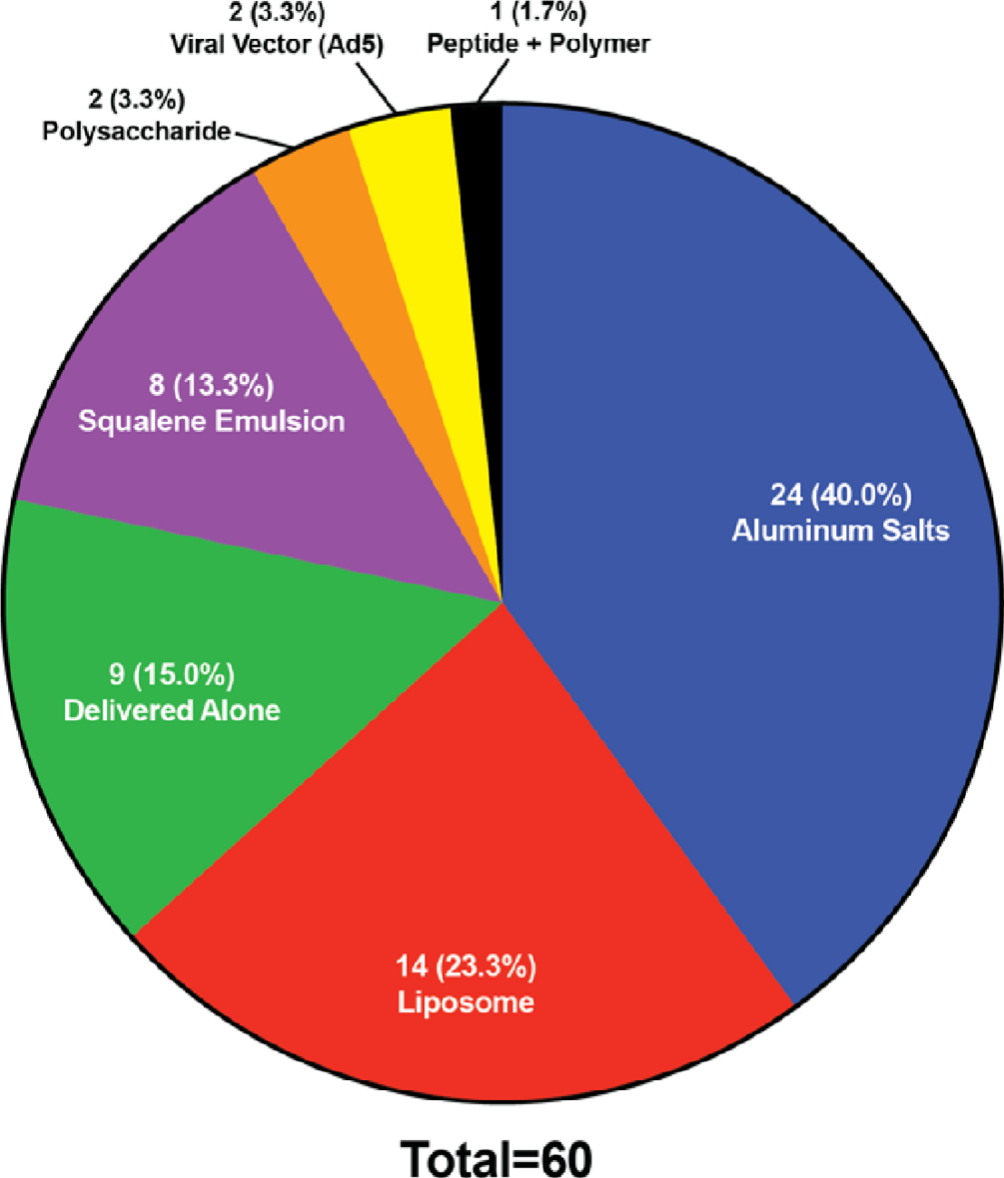
Make up of unique vaccine formulations that utilize a TLR agonist divided by the combination material it is formulated with. The number of unique vaccine formulations as well as the resulting percent of the whole space is provided.

As is seen in many of the trials evaluating TLR agonists, these TLR agonists are always not delivered alone; moreover, only 15% of the vaccine formulations utilized the TLR agonist alone with the antigen (Figure 6). Instead, these agonists are commonly combined with another adjuvant material to enhance not only their delivery and permanence at the injection site, but also the resulting immune response. The most common combination material utilized is aluminum salts and is in 40% of the vaccine formulations being evaluated in which the TLR agonist is adsorbed to the surface of the aluminum particle (Figure 6). Liposomes are the second most utilized combination material, employed in 23% of vaccine formulations containing a TLR agonist (Figure 6). All but one of these formulations specifically use a liposomal formulation of QS‐21 as seen in the adjuvant system AS01B, utilized in a licensed vaccine. As mentioned previously, QS‐21 is a fraction 21 saponin molecule extracted from the bark the South American tree *Quillaja saponaria* Molina. QS‐21 is an important immunomodulatory material that promotes a pro‐inflammatory environment and enhances the overall immune response to the antigen.^161^ Binding QS‐21 to cholesterol in a liposomal formulation is an important step in utilizing this material; free QS‐21 is toxic and causes necrosis of cells. However, when bound irreversibly to cholesterol, it loses its toxicity while maintaining adjuvanticity.^162^ While QS‐21 liposomal formulations vary in chemical composition and physical characteristics, the foundational materials utilized are consistent: phospholipids, cholesterol, QS‐21, and TLR agonist. Most commonly in the clinical trial space, liposomal formulations are combined with a TLR4 agonists, MPLA derivatives or synthetic analogs, with MA105 being the only liposomal formulation with a TLR3 agonist, Poly I:C. The AS01 system and ALFQ are the most prevalent liposomal adjuvants in the clinical trial space. AS01 utilizes a net neutral phospholipid, dioleoyl phosphatidylcholine (DOPC), with cholesterol in a 33.7 mol% ratio to the phospholipid while ALFQ employs two phospholipids, a neutral dimyristoyl phosphatidylcholine (DMPC) and anionic dimyristoyl phosphatidylglycerol (DMPG), with 55 mol% cholesterol. These chemical differences result in size differences across the two liposomes, with AS01 producing a homologous 100 nm particle and ALFQ resulting in heterogenous sizes of liposomes ranging from 50 to 30,000 nm.^163^ The liposome evaluated in clinic that does not contain QS‐21, ALF43, is a member of the ALF family, maintaining the same phospholipid composition and addition of 3D‐PHAD as ALFQ, but consisting of 43 mol% of cholesterol. Unlike aluminum salts, the only time liposomes, and specifically a liposomal formulation of QS‐21, appear in the current clinical trial space is when it is in combination with a TLR agonist.

Squalene emulsions are the third most common material combined with TLR agonists in the identified clinical trials. These appear in stable emulsions combined with GLA and SLA and will be discussed in the next section. An Ad5 vector which was discussed in the previous section is also utilized in the delivery of the dsRNA hairpin which functions as a TLR3 agonist. This combination appears in two unique vaccine formulations across three trials and is the only combination of a viral vector and TLR agonist in the clinical trial space. There are also novel combination materials represented in the clinical trial space. TriAdj is a unique combination of a host‐defense peptide IDR‐1002, a cationic polymer adjuvant polyphosphazene (PCEP), and the TLR3 agonist Poly I:C (NCT05693272). While IDR‐1002 has not displayed abilities to enhance pro‐inflammatory cytokine release from immune cells, it has shown in vitro to be a strong inducer of both neutrophil chemoattractant CXCL1 and CXCL8 and monocyte chemoattractant CCL2 and CCL7 on human peripheral blood mononuclear cells.^164^ This ability would aid in the creation of an immunocompetent environment at the injection site through leukocyte recruitment. PCEP on the other hand prompts the production of both IFNy and IL‐4 leading to a potent antigen‐specific Th1 and Th2 response when delivered in mice alone with antigen.^165^ Another novel combination material, dextran‐DEAE, is utilized in the GamTBvac vaccine against tuberculosis (NCT04975737). Dextran is a natural polysaccharide and one of the most studied α‐glucans in drug and antigen delivery; it has shown to enhance cellular and humoral responses, though the exact mechanism is unclear.^166^ In GamTBvac, a polycationic dextran derivatives, dextran‐DEAE, is surrounded by CpG 2216 (an TLR9 agonist) for the complete adjuvant system, while the antigens are fused with dextran‐binding domain and non‐covalently immobilized on dextran. Similarly to dextran, dextran‐DEAE's mechanism is not well understood, but it has been shown that this multicomponent adjuvant allows for slow release of antigen, stimulation of TLR9, and activation of phagocytosis all leading to a Th1 immune response.^167^ The final novel combination material is also a natural polysaccharide, inulin, which are formulated into particles and combined with the TLR9 agonist CpG55.2 for an adjuvant system used in the protection against COVID‐19 (NCT05279456). As a plant‐derived polysaccharide, inulin in its natural form has no immunomodulatory activity; however, when crystalized into particles forming delta inulin, it has potent adjuvant activity. Delta inulin is only soluble at temperatures above 40°C, meaning it remains stable in the body likely enhancing its immunological abilities. Importantly, it has also shown to initiate the human complement system leading to antibody responses as well as bind to monocytes and enhance their chemokine production.^168^ Each of these combination materials plays their own role in the adjuvant system, synergizing with the TLR agonist and enhancing the overall immune response.

### 3.5 Emulsion adjuvants

Emulsion adjuvants have been utilized in FDA‐licensed vaccines since 2013 when AS03 was utilized in the licensed H1N1 Vaccine followed by MF59 in 2015 in the influenza vaccine. Both AS03 and MF59 are squalene‐based oil‐in‐water emulsions with AS03 also including (d,l)‐α‐tocopherol (vitamin E) for additional immune activation. In the current clinical trial landscape, about 9% of the trials and about 8% of the unique vaccine formulations being evaluated contain an emulsion adjuvant (Figure 2). There are 12 unique emulsion adjuvants currently being evaluated in active trials, two of which (AS03 and MF59) are already utilized in licensed vaccines. Oil‐in‐water (O/W) emulsions are the most dominant form of emulsion adjuvants being tested in clinic, as well as in the licensed vaccine space, with 11 out of 12 of the unique emulsion adjuvants being oil‐in‐water emulsions. The other emulsion being evaluated is a water‐in‐oil (W/O) emulsion. The main difference between these two types of emulsions is their continuous and dispersed phase. O/W emulsions have a continuous phase of water with an oil phase dispersed throughout, while W/O emulsions are the reverse of this. Stability of the emulsion is difficult to achieve in both types of emulsions, but W/O emulsions have lower stability due to the high mobility of the water droplets.^169^ Additionally, toxicity and local reactogenicity is a larger concern with W/O emulsions due to the high concentration of oil, leading to less W/O emulsions being evaluated in clinic.^170^

#### 3.5.1 Water‐in‐oil emulsions

Montanide ISA‐51 is the only W/O emulsion currently being tested in clinical trials in formulation with peptides for protection against COVID‐19 (NCT04885361).^171^ This emulsion is composed of a mineral oil and surfactant from the mannide monooleate family.^172^ The W/O adjuvant will remain at the injection site and create a depot to release the antigen slowly over time which aids in the overall immune response. Additionally, this W/O depot induces inflammation and results in the recruitment of immune cells to take up the antigen.^173^ While these adjuvants are effective in enhancing the immune response to the antigen, they come with potentially severe local and systemic adverse effects, which has been the reason for the termination of related clinical trials in the past.^174^, ^175^ Specifically, when previously evaluated in an HIV vaccine, the injection of montanide ISA‐51 was followed by local swelling of the injection site as well as systemic responses such as headache, nausea, and fever.^174^ Of most concern was the formation of sterile abscesses at the injection site in multiple patients which is thought to be caused from adjuvant‐induced inflammation. The adjuvant can remain in the injection site for extended periods of time, with removal of the vaccine by immune cells taking months to complete. Due to this and the consistent engagement of TLRs from products in the oil prompting an inflammatory cascade, this adjuvant can result in severe inflammatory responses that negatively impact the patient.

#### 3.5.2 Oil‐in‐water emulsions

For O/W emulsions, squalene is a major material utilized in these adjuvants in clinical trials. In fact, of the 11 unique O/W emulsions in active trials, all but one includes squalene in their formulation (Table 2). Squalene is a triterpene that is naturally derived from a few key sources including plants and shark liver oil, with shark‐derived squalene being the most common in clinical adjuvant formulations.^176^ In many formulations, squalene is utilized to stabilize the formulation, facilitate solubilization of the antigen, and modify the release of the antigen. It is also a biocompatible material, which allows it to exhibit less sustained toxicity as compared to mineral oil utilized in many W/O emulsions. However, with overfishing decreasing the abundance of sharks and enhancing the protection around them, a more sustainably sourced resource is necessary in the upcoming years.^177^

**TABLE 2:** Composition of oil in water emulsions in clinical trials.

O/W emulsion	Unique formulations	Total trials	Buffer	Composition
GLA‐SE	7	9	Ammonium phosphate	Poloxamer 188 Glycerol 1,2‐dimyristoyl‐*sn*‐ glycero‐3‐phosphocholine (DMPC) Squalene TLR Agonist (GLA or SLA)
SLA‐SE	1	1		
MF59	7	7	Citrate	Tween 80 Span 85 Squalene
SCT‐VA02B	3	15		
SWE	1	2		
SQBA	1	1		
AS03	5	11	PBS	Tween 80 (d,l)‐α‐tocopherol Squalene or squalane
BFA03	1	2		
Squalane‐based emulsion	1	1		
LiteVax	1	1	PBS	Tween 80 CMS (Maltose 4′‐monosulfate 1,2,3,6,2′,3′,6′‐heptadecanoic acid ester) Squalene
Ne01 (20% Nanoemulsion)	1	1	Water	Tween 80 Ethanol Cetylpyridinium chloride Soybean oil

##### MF59 similars

The licensed squalene emulsion adjuvant, MF59, is currently being evaluated in seven clinical trials for influenza and COVID‐19 vaccines. Three other adjuvants in the current clinical trial space utilize the same components as MF59 (Table 2), with SCT‐VA02B employing the same formulation in three unique vaccine formulations against COVID‐19.^178^ SWE formulates the emulsion with slightly altered composition and is therefore free for technology transfer to equip vaccine manufacturers in developing countries; its composition has squalene representing 3.9%, and both surfactants at 0.47%, and is currently being evaluated in a COVID‐19 vaccine (NCT05209009).^179^ The SQBA adjuvant presents a similar formulation to SWE and is also being evaluated in a COVID‐19 vaccine (NCT05142514).^180^ Despite the slight variations in formulation, these squalene emulsions utilize the same immune activation and responses to function effectively as adjuvants. They create a transient and local immune‐competent environment at the injection site through ATP release from muscle cells and cytokine induction that leads to the influx of inflammatory cells to take up antigen and carry it to the draining lymph node for processing.^181^, ^182^ MF59 has also been shown to induce RIPK3‐dependent necroptosis followed by apoptosis in the lymph node, which has been connected to its ability to induce CD8+ T‐cell responses and antibody responses, respectively.^181^ The impacts of each individual component in strengthening the immune response have been investigated for MF59. Individual components are not as potent as the formulation as a whole.^20^ In fact, squalene, Tween 80, and citrate buffer had no immune stimulatory effects when delivered alone. Span 85 did have a stimulatory effect in the muscle, but did not lead to antibody or T‐cell generation which the MF59 formulation accomplishes.^20^

##### AS03 similars

AS03 is another squalene‐based emulsion, with slightly different composition through the inclusion of DL‐α‐tocopherol and removal of Span 85, all formulated in a PBS buffer. Two other adjuvants currently evaluated in clinic contain distinct variants of AS03 (Table 2), with BFA03 representing a similar formulation of the same components and squalane‐based emulsion formulated with less DL‐α‐tocopherol (10 mg/ml) and a saturated derivative squalane (30 mg/ml).^183^, ^184^ AS03 is currently in five unique vaccine formulations across 11 trials for the protection against COVID‐19 and Hepatitis B, with BFA03 (NCT05398848) and squalane‐based emulsion (NCT05726084) each in one unique vaccine formulation for the protection against COVID‐19. The addition of DL‐α‐tocopherol, which is a synthetic form of vitamin E, differentiates these formulations and their resulting immune responses from those akin to MF59. Similar to squalene, DL‐α‐tocopherol is a biodegradable polyprenyl. When compared to an AS03 formulation without DL‐α‐tocopherol, in which an equivalent amount of squalene was used as replacement, the formulation without DL‐α‐tocopherol had an earlier increase in cytokine release in the muscle, compared to the complete formulation which modulated more proinflammatory cytokines overall and at 24 h after injection. Additionally, when DL‐α‐tocopherol was omitted from formulation, a decrease of granulocyte infiltration into the draining lymph node and antigen‐loading of monocytes was observed. These formulation‐dependent changes of the innate immune response resulted in a decrease in antibody responses by 3.5‐ to 6‐fold when vaccinating without DL‐α‐tocopherol versus with this component in formulation.^16^ Overall, AS03 and its similars currently in clinic benefit from the addition of DL‐α‐tocopherol. When compared directly to MF59 in protection against an H5N1 strain, both adjuvants were able to elicit broad homo‐ and heterosubtypic antibody responses; however, AS03 achieves both a higher titer and breadth of antibody responses compared to MF59, highlighting the potential benefits of DL‐α‐tocopherol in formulation.^185^

##### GLA‐SE and SLA‐SE

Both GLA‐SE and SLA‐SE utilize the same squalene emulsion called stable emulsion (SE) that allows for the absorption of TLR agonists GLA and SLA, respectively. The emulsions are developed by AAHI, who also developed the TLR agonists. These emulsions were initially designed firstly on stability of the formulation and secondly on the resulting adaptive immune response.^186^ The resulting emulsion consisted of mixing and emulsifying an aqueous phase in ammonium phosphate buffer with the surfactant poloxamer 188 (0.9 mg/ml) and glycerol (22.5 mg/ml) with a 10% oil phase consisting of DMPC (19 mg/ml) and GLA or SLA (0.25 mg/ml) in squalene oil.^187^ This particle of 75–90 nm in size was found to induce formulation‐dependent immune responses in mice. Specifically, when evaluating GLA‐SE, SE alone, and GLA in an aqueous formulation (GLA‐AF), GLA‐SE was shown to produce a robust antigen‐specific Th1 response compared to the other formulations, with an increase in antigen‐specific Th1 CD4 cells and antibody isotype switching to the IgG2c subclass. This enhancement of innate and adaptive responses has been largely tied to GLA‐SE's ability to initiate IFNy production and engage the inflammasome—a pathogen recognition pathway—as compared to SE alone or GLA‐AF.^188^ In the current clinical trial space, SLA‐SE is utilized in one trial for the protection against herpes zoster (NCT05304351) while GLA‐SE is as predominant as MF59 in the space appearing in seven unique vaccine formulations across nine trials in total, making it the most studied emulsion formulation in active clinical trials that has not been utilized in FDA‐licensed vaccines.

##### LiteVax

LiteVax is a novel squalene emulsion formulation that maintains similarity to MF59 and AS03 with squalene being the main component at 80 mg/ml and the surfactant Tween 80 at 40 mg/ml. Currently in the clinic, LiteVax is being utilized as the adjuvant for the licensed seasonal flu vaccine (NCT05581407). LiteVax differs from both AS03 and MF59 in the introduction of an additional immunomodulatory component, maltose 4′‐monosulfate 1,2,3,6,2′,3′,6′‐heptadecanoic acid ester (CMS) at 40 mg/ml, formulated with the other components in PBS. CMS is a monosulfate derivative of a carbohydrate fatty acid sulfate ester (CFASE). CFASE adjuvants were explored previously in cancer vaccines and as adjuvants for infectious diseases such as influenza; however, its toxicity and side effects, such as local reactogenicity and body temperature increases, resulted in modifications of CFASE being explored.^189^, ^190^, ^191^ CMS is less reactogenic than CFASE, while maintaining immunological benefits and being able to produce antibody titers 10–20 fold higher than an MF59 similar when vaccinating against influenza.^191^ While the complete LiteVax formulation was found to mildly modulate dendritic cell activation in vitro, CMS alone was found to be more effective in upregulating costimulatory markers for maturation than the complete formulation.^192^ This indicates the importance of CMS for the immunomodulatory activity of LiteVax, and the potential of CMS to enhance immune responses to emulsion adjuvants.

##### Ne01 (20% nanoemulsion)

Ne01 is the only O/W emulsion in active clinical studies that does not include squalene in the formulation (Table 2). It is specifically designed to function as a mucosal adjuvant and is currently being evaluated for an intranasal H5N1 vaccine (NCT05397119). This emulsion (400 nm in size) was originally invented at the University of Michigan and is now developed by BlueWillow Biologics. It is manufactured by the emulsification of cetyl pyridinium chloride (CPC, 1%), Tween 80 (5%) and ethanol (8%) in water with soybean oil (64%) and remains stable and immunogenic in combination with antigen for up to 6 months at room temperature and 3 months at 40°C.^193^ This adjuvant has shown to produce strong cellular and humoral responses in ferrets and effectively protects them from lethal challenge of H5N1.^194^ While the exact mechanism of this adjuvant is still being elucidated, it has shown to create a Th17 response through the production of IL‐6 and IL‐17 and also a Th1 response through the production of IFNy.^195^ Additionally, it was shown that Ne01 results in antigen‐uptake and subsequent apoptosis of epithelial cells which are then engulfed by dendritic cells, resulting in an indirect route of antigen uptake and APC maturation.^196^

### 3.6 Previously licensed live‐attenuated vaccines

Live‐attenuated vaccines have inherent adjuvanticity due to the utilization of live, weakened viruses. In a clinical trial for the protection against malaria, both BCG – the live attenuated tuberculosis (TB) vaccine—and YF‐17D – the live attenuated yellow fever vaccine also known as Stamaril—are injected intradermally along with the antigen for use as an adjuvant (NCT05468606). BCG, or Bacillus Calmette‐Guerin, was developed by Calmette and Guerin and was first administered to humans in 1921 and remains the only licensed TB vaccine to date. It consists of a live‐attenuated strain of *Mycobacterium bovis*, the causative agent for TB in cattle, and has been shown to offer protection against other mycobacterial infections such as leprosy and Buruli ulcer in addition to TB.^197^ It is considered the world's most widely used vaccine, with approval by the League of Nations—now World Health Organization (WHO)—in 1928 and still included in WHO's Expanded Program on Immunization in 1976.^198^ The BCG vaccine broadly induces a proinflammatory response at the injection site leading to an increase in immune cell infiltration and IFNy release. Various proteins expressed by BCG function as TLR agonists and can lead to stimulation of immune cells. Ultimately these innate responses result in a strong humoral and cellular adaptive immune response.^199^ The yellow fever vaccine, Stamaril or YF‐17D, was prequalified by the WHO in 1987 yet is still considered investigational by the FDA and is not a licensed product in the US. Developed by Sanofi, it utilizes the live attenuated 17D‐204 strain of the yellow fever virus; importantly, the 17D line of yellow fever virus vaccines are considered to be among the most effective and safest live‐attenuated vaccines ever produced.^200^ The vaccine activates several dendritic cell subsets through multiple TLR agonists resulting in the production of pro‐inflammatory cytokines and leading to a robust humoral and cellular adaptive immune response.^201^

## 4 CONCLUSIONS

Adjuvants are an important component of vaccines that can not only enhance the overall immune response but also enhance vaccine stability. When utilized in vaccines against infectious diseases, applying materials that can allow vaccines to be transported to areas without effective refrigeration or decreasing the cost of the vaccine through reducing the required vaccine dose and/or frequency is of upmost importance. Yet, even with the robust potential of adjuvants to improve vaccination, it took nearly 80 years for the second adjuvant to receive FDA‐approval after aluminum salts' approval. While there has been more adjuvants utilized in licensed vaccines since the 2010s, aluminum salts continue to dominate the adjuvant space and remain the most utilized adjuvant in the clinical trial space. However, just like a single antigen cannot be expected to confer protection to all pathogens, neither can an adjuvant. As experienced during the COVID‐19 pandemic, new or complex viruses and pathogens require intentionality of design in the adjuvant space as well as the antigens. This complete vaccine design is important to elicit broad and long‐term protection. While over half of the active clinical trials for infectious disease vaccines did not use an adjuvant or used one in a previously licensed vaccine, 36% of the trials are investigating newer adjuvants that are not yet utilized in the licensed vaccine space. These adjuvants are based on various materials such as nanoparticles, viral vectors, TLR agonists, and emulsions, and are used alone or in combination with other immunomodulatory materials.

Looking ahead, more materials should be evaluated as adjuvants in vaccination and their immunomodulatory effects should be evaluated for multiple antigens and diseases. There is a clear gap in the clinical trial space in adjuvant innovation and exploration. We cannot continue to rely on materials we already have to meet the needs to the upcoming pandemics or expect a poorly immunogenic antigen to induce a strong enough immune response. With a more robust materialistic understanding of immunomodulatory compounds, we can be prepared to formulate efficacious vaccines involving rationally designed antigens as well as adjuvants. The safety of adjuvants is also of extreme importance when vaccinating the general public, and probably can be looked to as a major reason for why a large number of vaccines evaluated in clinical trials don't use an adjuvant or involve one from a previously licensed vaccine, with validated safety profiles. However, by designing adjuvants intentionally with safety and immunogenicity in mind, we can ultimately formulate more effective vaccines that do not sacrifice safety of the patient. While vaccines have long been a part of public health, the field of adjuvants is in relative infancy, and it will be exciting to see the new materials we welcome into the space in the future.

## AUTHOR CONTRIBUTIONS

**Morgan Goetz:** Conceptualization; data curation; formal analysis; methodology; writing – original draft; writing – review and editing. **Naaz Thotathil:** Data curation; formal analysis; investigation; methodology; writing – original draft. **Zongmin Zhao:** Writing – review and editing. **Samir Mitragotri:** Conceptualization; funding acquisition; supervision; writing – review and editing.

## CONFLICT OF INTEREST STATEMENT

Authors declare no conflict of interest.

### PEER REVIEW

The peer review history for this article is available at https://www.webofscience.com/api/gateway/wos/peer-review/10.1002/btm2.10663.

## Data Availability

The data that support the findings of this study are available from the corresponding author upon reasonable request.
